# Amplification of MUC1 in prostate cancer metastasis and CRPC development

**DOI:** 10.18632/oncotarget.13073

**Published:** 2016-11-04

**Authors:** Nicholas Wong, Pierre Major, Anil Kapoor, Fengxiang Wei, Judy Yan, Tariq Aziz, Mingxing Zheng, Dulitha Jayasekera, Jean-Claude Cutz, Mathilda Jing Chow, Damu Tang

**Affiliations:** ^1^ Division of Nephrology, Department of Medicine, McMaster University, Hamilton, Ontario, Canada; ^2^ Father Sean O'sullivan Research Institute, Hamilton, Ontario, Canada; ^3^ The Hamilton Center for Kidney Research, St. Joseph's Hospital, Hamilton, Ontario, Canada; ^4^ Division of Medical Oncology, Department of Oncology, McMaster University, Hamilton, Ontario, Canada; ^5^ Department of Surgery, McMaster University, Hamilton, Ontario, Canada; ^6^ The Genetics Laboratory, Longgang District Maternity and Child Healthcare Hospital, Longgang District, Shenzhen, Guangdong, P.R. China; ^7^ Department of Pathology and Molecular Medicine, McMaster University, Hamilton, Ontario, Canada; ^8^ Department of Respiratory Medicine, Shenzhen 2nd People's Hospital, Shenzhen, Guangdong, China; ^9^ Department of Respiratory Disease, The First Affiliated Hospital of Shenzhen University Medical School, Shenzhen, Guangdong, China

**Keywords:** MUC1, prostate cancer, prostate cancer stem cells, metastasis, castration resistant prostate cancer

## Abstract

Evidence supports the upregulation of MUC1 in prostate cancer (PC). However, this has not been thoroughly investigated. We report here an association of MUC1 upregulation with PC metastasis and the development of castration resistant PC (CRPC). MUC1 expression was specifically increased in DU145 cell-derived PC stem-like cells (PCSLCs) in comparison to their non-PCSLCs counterparts. While immunohistochemistry staining of 34 primary PCs revealed variability in MUC1 expression, Nanostring technology demonstrated elevated MUC1 mRNA levels in 4 of 7 PCs compared to their normal matched tissues. By analyzing MUC1 mRNA levels and gene copy number (GCN) using the Oncomine^TM^ database, elevations in MUC1 mRNA in 82 metastases versus 280 primary PCs and in MUC1 GCN in 37 metastases over 181 primary tumors were demonstrated. Analysis of genomic datasets within cBioPortal revealed increases in MUC1 GCN in 2% (6/333) of primary PCs, 6% (9/150) of metastatic PCs, and 33% (27/82) of CRPCs; in comparison, the respective increase in androgen receptor (AR) GCN was 1%, 63%, and 56%, revealing a specific increase in MUC1 GCN for CRPC. Furthermore, a 25-gene MUC1 network was amplified in 52% of CRPCs compared to 69% of CRPCs displaying increases in an AR co-regulator group. While genomic alterations in the MUC1 network largely overlap with those in the AR group, 18 CRPCs (66.7% being neuroendocrine PC) showed genomic alterations only in the MUC1 network. Moreover, genomic alterations in the MUC1 network correlated with PC relapse. Collectively, our observations suggest a combination therapy involving MUC1-based immunotherapy and androgen deprivation.

## INTRODUCTION

Prostate cancer (PC) is the most prevalent male-specific cancer in the developed world [1]. PC progresses from high grade prostatic intra-epithelial neoplasia (HGPIN), to local carcinoma, to metastatic disease with bone as the preferential site [2]. Localized tumors can be effectively managed through a variety of approaches, including watchful waiting, surgical removal, and radiation. In contrast, options for patients with metastatic PC remain limited. Androgen deprivation therapy (ADT), a strategy that was pioneered by Charles Huggins in the 1940s [3, 4], remains the standard of care in these patients. However, the treatment is only palliative, as resistant tumors in the form of castration resistant PC (CRPC) inevitably arise. Until recently, these patients were commonly treated with docetaxel-based chemotherapy. Cumulative research efforts have revealed the dependency of androgen receptor (AR) signalling despite androgen deprivation for a large proportion of CRPCs [5, 6], which led to the recent development of abiraterone and enzalutamide for treatment [7, 8]. Additionally, the cell-based vaccine Sipuleucel T has recently become available [9, 10], a therapy that depends on tumor associated antigens (TAAs).

Mucin 1 (MUC1) is the most well-characterized TAA [11]. The glycoprotein is a transmembrane member of the mucin family, and is broadly expressed on the apical surface of most epithelial tissues, including the pancreas, breast, lung, and gastrointestinal tract [11, 12]. MUC1 is a heterodimer consisting of a large N-terminal fragment (MUC1-N) that is anchored to the cell membrane on the extracellular side by binding to the transmembrane C-terminal MUC1 subunit (MUC1-C). Mature MUC1 is formed from auto-cleavage of a pre-peptide [13–15]. MUC1-N contains a variable number of conserved tandem repeats of 20 amino acids, which are highly glycosylated by *O*-linked glycans. The MUC1 protein is expressed on the apical surface of epithelium and plays a protective role for the mucosal epithelial surface [16]. However, it is aberrantly expressed in numerous malignancies with respect to loss of polarity in cancer cells, overexpressed in over 70% of cancers, and differentially glycosated [11, 17]. The cancer-associated MUC1 with aberrant glycosation is highly immunogenic [18–20]. These properties have made the MUC1 TAA a major focus in developing antigen-specific immunotherapies for multiple tumor types [12].

Our recent phase I/II clinical trial using a Tn-MUC1 peptide-based cell vaccine (dendritic cells/DC) revealed that this approach was able to delay the doubling of prostate specific antigen (PSA) levels in CRPC patients, demonstrating utility in developing MUC1-based DC vaccines in treating this population [21]. However there are mixed messages, depending on which antibody is used, regarding the detection of MUC1 overexpression in PC progression. Increases in the MUC1 protein and aberrant MUC1 glycosation were reported in PC [22–24]. However, using a different antibody (anti-MUC1-N, HMFG2), increased MUC1 expression in PC progression could not be demonstrated [25]. To investigate the association of MUC1 expression with PC tumorigenesis, we have made our own effort to track MUC1 though PC progression. In our immunohistochemical examination of MUC1 expression, we were also unable to show a MUC1 increase in PCs with Gleason scores (GS) ≥8 in comparison to those with GS6-7. We reasoned that the differences among individual investigations might be attributable to the antibodies used, recognizing MUC1-N which can be shed off from the cell surface [17]. With this in mind, we used an alternative approach and have examined increases in MUC1 mRNA and gene copy number (GCN) following PC progression. With this effort, we clearly demonstrate elevated MUC1 mRNA levels in metastatic PCs and amplification of the MUC1 gene in CRPCs.

## RESULTS

### Association of MUC1 with prostate cancer stem-like cells (PCSLCs) and PC progression

**Figure 1:**
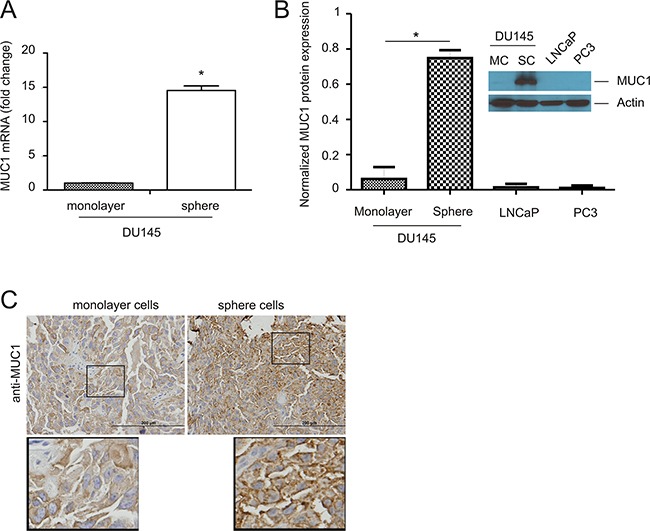
Upregulation of MUC1 in prostate cancer stem-like cells (PCSLCs) **A.** Real-time PCR analysis of MUC1 mRNA in the indicated PC cell lines. β-actin was used as an internal control. Experiments were repeated three times. MUC1 mRNA abundance is graphed as a fold change to monolayer cells; mean±SD (standard deviation) are graphed. *:p<0.05 by a 2-tailed Student's *t*-test. **B.** MUC1 protein expression in DU145 monolayer cells (MC) and sphere cells (SC), LNCaP, and PC3 cells was determined by Western blot. Experiments were repeated three times; typical images from a single repeat are shown (inset). MUC1 protein levels were normalized to the respective actin; mean±SD are graphed. *p<0.05 by a 2-tailed Student's *t*-test in comparison to DU145 monolayer cells. **C.** MUC1 expression in xenograft tumors produced by DU145 monolayer or sphere cells was determined by immunohistochemistry (IHC). The indicated areas are enlarged three fold and placed underneath the original panel.

There is accumulating evidence demonstrating an association of MUC1 upregulation with cancer progression [26], including PC [27]. It is also clear that cancer consists of heterogeneous cell populations [28–30], in which cancer stem cells (CSCs) are critical for cancer progression [31–33]. In accordance with this concept, MUC1 has previously been reported to associate with and contribute to the development of breast cancer stem cells [34, 35]. Similarly, prostate cancer stem cells (PCSCs) are essential for PC progression [36], suggesting a relationship between MUC1 and PCSCs. By taking advantage of our recently established PCSLCs (sphere cells) derived from DU145 monolayer cells [37], we have demonstrated a significant upregulation of MUC1 in DU145 sphere cells at both mRNA and protein levels (Figure 1A, 1B). Prolonged film exposure also depicts low MUC1 protein expression in DU145 monolayer, PC3, and LNCaP cells (data not shown). Low levels of the MUC1 protein in a set of non-PCSLCs (DU145, PC3, and LNCaP) is consistent with their reduced MUC1 mRNA levels (Supplementary Figure S1); decreased MUC1 mRNA expression was also observed in 22Rv1 PC cells and immortalized human prostate epithelial BPH1 cells (Supplementary Figure S1). Since the real time primers amplified a MUC1-C region which is present in MUC1, MUC1 splice variants, MUC1/Z, and MUC1/Y [38], the data presented in Supplementary Figure S1 support a general and significant elevation of MUC1 expression in DU145 cell-derived PCSLCs in comparison to their non-PCSLC counterparts and multiple other non-PCSLCs (Supplementary Figure S1). Furthermore, an increase in MUC1 was also demonstrated in xenograft tumors generated from DU145 sphere cells compared to those produced by DU145 monolayer cells (Figure 1C). Taken together, the above observations reveal an association of MUC1 with PCSLCs.

**Figure 2:**
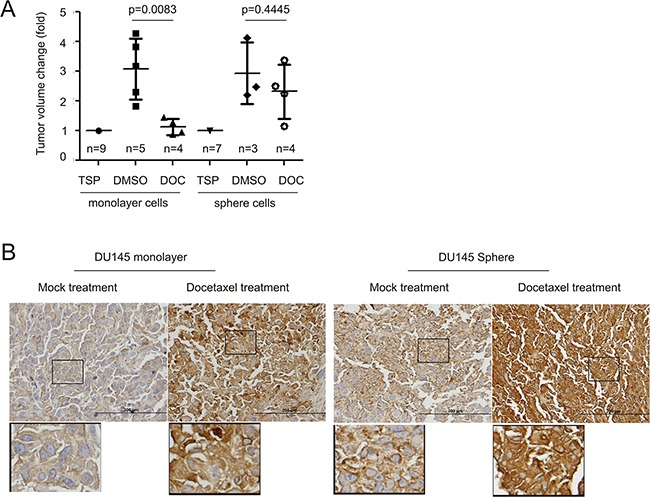
Docetaxel upregulates MUC1 expression in xenograft tumors **A.** DU145 monolayer (10^6^) or sphere (10^4^) cells were subcutaneously implanted into 9 and 7 NOD/SCID mice, respectively. When tumors reached 100mm^3^ (treatment starting point/TSP), mice were randomly assigned to receive DMSO or docetaxel (see Materials and Methods for details). Tumor volumes are expressed as fold change two weeks after the TSP. Statistical analyses were performed using Student's *t*-test. **B.** IHC staining for MUC1 in xenograft tumors generated from DU145 monolayer and sphere cells. Typical images are shown. The indicated regions are enlarged three fold and placed underneath the original panel.

We have previously demonstrated that DU145 sphere cells possess a 100-fold higher capability of tumorigenesis [37] and are more resistant to a genotoxic reagent-induced cytotoxicity [39] in comparison to DU145 monolayer cells, further suggesting an association of MUC1 upregulation with chemoresistance in PC. To examine this possibility, we produced xenograft tumors from DU145 monolayer and sphere cells. Docetaxel treatment for 2 weeks significantly reduced tumor volume for monolayer cell-derived xenografts but not for sphere cell-derived xenografts, as expected of PCSLCs (Figure 2A). Upon analysis, the latter expressed elevated levels of MUC1 in comparison (Figure 2B, mock treatment). Intriguingly, docetaxel treatment increased MUC1 expression in xenograft tumors produced by both cell types. Sphere cell-derived xenografts displayed a robust elevation of MUC1 in response to docetaxel when compared to monolayer cell-derived tumors (Figure 2B). These observations are in line with a recent report demonstrating that docetaxel treatment increased MUC1 expression in LNCaP cell-derived xenograft tumors [40]. Collectively, our study supports an association of MUC1 upregulation with PC progression.

### MUC1 increases in metastatic PCs

**Figure 3:**
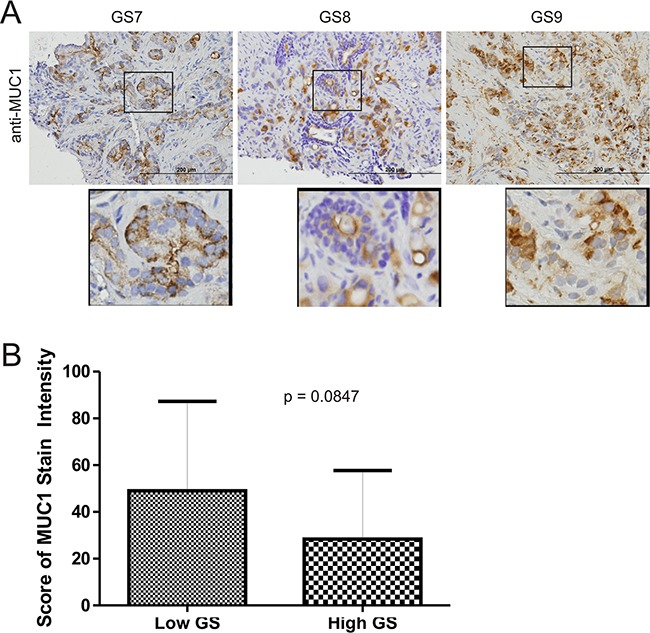
Detection of MUC1 in primary prostate cancers **A.** IHC staining for MUC1 in 34 primary PC tissues (Table 1). Typical images for Gleason score (GS) 7, 8, and 9 tumors are shown. Scale bars represent 200μm. **B.** IHC staining was quantified through ImageScope software (see Materials and Methods for details). Average HScores±SDs are included in Table 1. The averaged scores±SDs for low Gleason score (GS6-7) and high Gleason score (GS8-10) tissues are graphed. Statistical analyses were performed using Student's *t*-test.

To further study the above association, we have examined MUC1 protein expression in 34 primary PCs consisting of 13 low (GS6-7) and 21 high grade PCs (GS8-10) (Table 1). By using an anti-MUC1-N antibody (BD), MUC1 presence was clearly detected in PC tumors (Figure 3A), but with variable levels (Table 1). MUC1 expression was not greater in high grade PCs than low grade PCs (Figure 3B) as we had expected. Tumor cells can shed off MUC1-N [17], making this a possibility for PCs without detectable staining. To test this scenario, we examined 14 primary PC slides from our PC cohort (Table 1) and an additional 6 primary PCs side-by-side using an anti-N or anti C-terminus antibody. For staining with either antibody, we observed PCs with 1) high intensive staining for both antibodies; and 2) importantly, a low level of staining for MUC1-N but intensive staining for MUC1-C (Supplementary Table S1, Supplementary Figure S2). These observations thus support that the lack of detectable staining for the N-terminal fragment is due to potential shedding of MUC1-N.

**Table 1:** Patient information and MUC1 score

Patient #	Age^a^	Gleason Score	Metastasis	Average Score^b^
1	76	3+3	No	51.79± 19.76
2	55	3+3	No	44.14± 16.54
3	53	3+3	No	22.55± 12.72
4	52	3+3	No	39.77± 18.04
5	58	3+3	No	130.63± 98.65
6	59	3+3	No	8.20± 6.48
7	50	3+4	No	37.03± 5.04
8	69	3+4	No	113.14± 32.31
9	70	3+4	No	43.76± 15.42
10	78	3+4	No	24.32± 15.95
11	70	3+4	No	3.64± 4.08
12	50	4+3	No	34.01± 18.05
13	74	4+3	No	84.61± 38.31
14	79	4+4	No	16.81± 4.97
15	56	4+4	Yes^c^	18.76± 24.62
16	58	4+4	Yes^c^	57.89± 26.93
17	60	4+4	No	12.19± 10.54
18	75	4+5	No	13.35± 3.45
19	82	4+5	No	6.19± 5.83
20	72	4+5	No	38.36± 7.09
21	55	4+5	No	11.41± 2.93
22	49	4+5	No	11.61± 8.4
23	64	4+5	No	4.24± 4.7
24	71	4+5	No	4.22± 5.14
25	65	4+5	No	10.06± 6.15
26	81	4+5	No	7.99± 14.96
27	89	5+4	No	69.16± 29.67
28	77	5+4	No	43.00± 18.93
29	65	5+4	Yes^d^	1.73± 0.73
30	80	5+4	No	57.85± 22.02
31	74	5+5	No	62.09± 26.33
32	68	5+5	No	34.78± 47.69
33	91	5+5	No	0.58± 0.96
34	98	5+5	No	112.84± 14.86

a age at diagnosis

b average score of stain intensity ± SD

c patient also demonstrates metastasis to lymph node

To compensate for the limitation of using anti-MUC1 antibodies in PC tissues, we went on to investigate MUC1 mRNA in 7 pairs of PC and benign prostate tissues using state-of-the-art Nanostring technology. The PC tissues contained 60-80% carcinoma. This was validated by demonstrating PTEN downregulation and ERG upregulation (indicative of TMPRSS2-ERG fusion) in 5/7 of the PC tissues (Table 2). In comparison to their respective benign prostate tissues, MUC1 mRNA was elevated in 4 PC cases (Table 2, p=0.044), supporting the notion of MUC1 upregulation in PC.

**Table 2:** Nanostring analysis of gene expression in primary prostate cancer tissues

Genes	P1^a^	P2^a^	P3^a^	P4^a^	P5^a^	P6^a^	P7^a^
MUC1	+2	+2.4	N^c^	+3.1	N	+1.4	N
TMPRSS2-ERG^b^	+16.2	+30.2	N	N	+17	+25.3	+27.3
PTEN^b^	−1.4	−1.4	N	N	−1.4	−1.3	−2.6

a patients 1-3 (GS6), patients 4-6 (GS7, 4+3 for P4,5, and 3+4 for P6), and P7 (GS4+4).

b TRPRSS2-ERG and PTEN were used as positive controls for upregulated and downregulated genes in PC.

c no downregulation or upregulation

Student's t-test (2-tails) was used to determine the p-values for changes in MUC1 (p=0.044), PTEN (p=0.051), and ERG (p=0.004).

**Figure 4:**
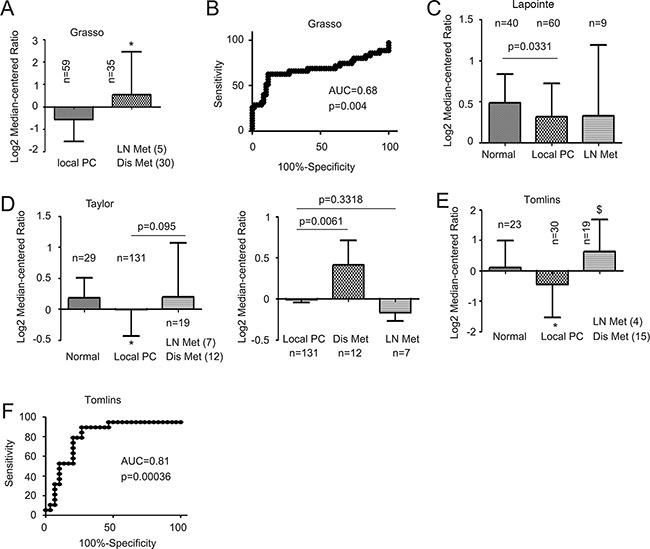
Upregulation of MUC1 mRNA in metastatic PC Data were extracted from the Grasso **A, B.** Lapointe **C.**, Taylor **D.**, and Tomlins **E, F.** datasets from the Oncomine^TM^ database (Compendia Bioscience, Ann Arbor, MI), and analyzed for changes in MUC1 mRNA. Mean±SD are graphed (A, C, D, E). (B, F) A receiver-operating characteristic (ROC) curve of local versus metastatic PC was derived from the data extracted from the Grasso (B) and Tomlins datasets (D). AUC: area under the curve. LN Met (4): lymph node metastasis (n=4); Dis Met (12): distant metastasis (n=12).

To thoroughly investigate the relationship of MUC1 mRNA levels and PC progression, we extracted MUC1 mRNA data from four datasets in the Oncomine^TM^ database (Compendia Bioscience, Ann Arbor, MI); the Grasso, Lapointe, Taylor, and Tomlins datasets [27, 41–43]. In the analysis of MUC1 mRNA in 221 PCs (60+131+30) and 92 normal prostate tissues (40+29+23), MUC1 mRNA was actually reduced in primary PCs (Figure 4C, D, and E). However, an increase in MUC1 mRNA was observed in metastatic PCs compared to primary tumors in the Grasso (Figure 4A) and Tomlins datasets (Figure 4E). This difference is particularly clear between primary and distant metastasis (Figure 4D, right panel). Additionally, this elevation differentiated metastases from organ-confined tumors according to ROC curves (Figure 4B and 4F). Collectively, these analyses suggest a general increase in MUC1 mRNA following PC metastatic progression.

**Figure 5:**
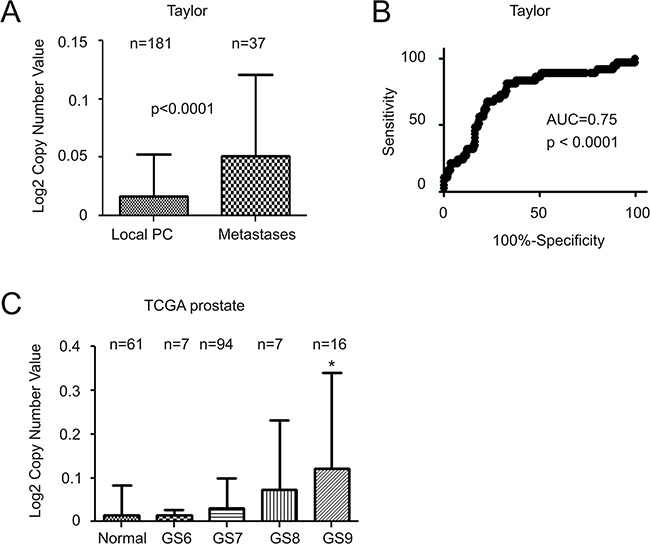
Increases in MUC1 gene copy number in advanced and metastatic PC Data related to MUC1 gene copy number was extracted from the Taylor **A, B.** and TCGA (generated by the TCGA Research network, http://cancergenome.nih.gov/) datasets from Oncomine^TM^ (Compendia Bioscience, Ann Arbor, MI); mean±SD **A** and **C.** and a ROC curve of primary versus metastatic PC were calculated and graphed. Statistical analyses were performed using Student's *t*-test. AUC: area under the curve.

The above concept is supported by increases in MUC1 gene copy number (GCN) in metastatic PCs compared to primary tumors (Figure 5A). Once again, this increase is able to separate metastases from primary PC via an area under curve (AUC) value of 0.75 (Figure 5B). Lastly, among 124 PC tumors and 61 benign prostate tissues analyzed in the TCGA dataset, a significant increase in MUC1 GCN could only be demonstrated in GS9 PCs compared to benign prostate tissues (Figure 5C). Taken together, these results support MUC1 upregulation as being a late event during PC progression and in metastatic cases.

**Figure 6:**
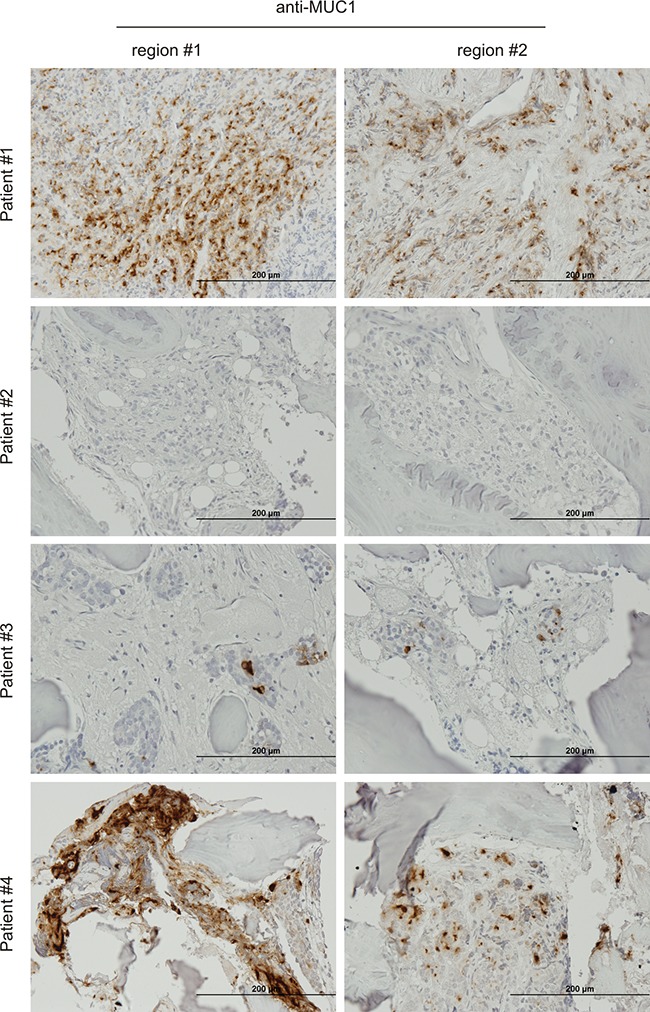
Expression of MUC1 in bone PC metastases IHC staining of four PC bone metastases. Two typical regions from each patient are shown. The overall staining (low magnification) is presented in Supplementary Figure S3.

Finally, we also made an effort to examine MUC1 protein in PC bone metastasis. In four bone metastases examined, MUC1 was detected heterogeneously among patients, in different tumor masses within the same tissue, and among different cancer cells within the same tumor mass (Figure 6, Supplementary Figure S3). We could not detect MUC1 expression in patient #2 (Figure 6, Supplementary Figure S3), which might be attributable to a relatively low level of tumor load (Supplementary Figure S3). For the other three patients, while the number of cells expressing MUC1 varied, its presence was readily detected in positive cells (Figure 6). The heterogeneous detection of MUC1 (Figure 6, Supplementary Figure S3) is unlikely attributed to use of an anti-MUC1-N antibody; anti-MUC1-C antibody did not produce any detectable staining in all four bone metastases (data not shown), an issue that needs further investigation. This pattern of heterogeneous expression is consistent with MUC1's association with our prostate cancer stem-like cells. Although it is impossible to compare MUC1 protein expression in our limited number of bone metastases to local PCs, bone metastases clearly express MUC1 (Figure 6).

### Specific amplification of the MUC1 gene in CRPC

Advances in next-generation (second-generation) sequencing (NGS) technologies have made whole genome sequencing a reality. There are currently 10 datasets of genome sequencing studies deposited into the cBioPortal database [44, 45], and these resources cover primary, metastatic, and castration resistant PC (cBioPortal/ http://www.cbioportal.org/index.do). By taking advantage of this rich source of PC genomic data, we have systemically analyzed alterations in MUC1 GCN. Among 333 prostate adenocarcinomas [46], 150 metastatic PCs [47], and 107 CRPCs [48], MUC1 gene is amplified in 1.8%, 6%, and 30% of the patient cohorts, respectively (Table 3), demonstrating a unique amplification of the MUC1 gene in CRPCs.

**Table 3:** MUC1 gene copy amplification in prostate cancer^a^

PC type	Cases^b^	Amp cases^c^	%	references
CRPC	77	27	35	48
Metastatic PC	150	9	6	47
Local PC	333	6	1.8	46

a data was extracted from cBioportal for Cancer Genome

b total number of PC cases

c the number of cases with the MUC1 gene copy number amplification

**Figure 7:**
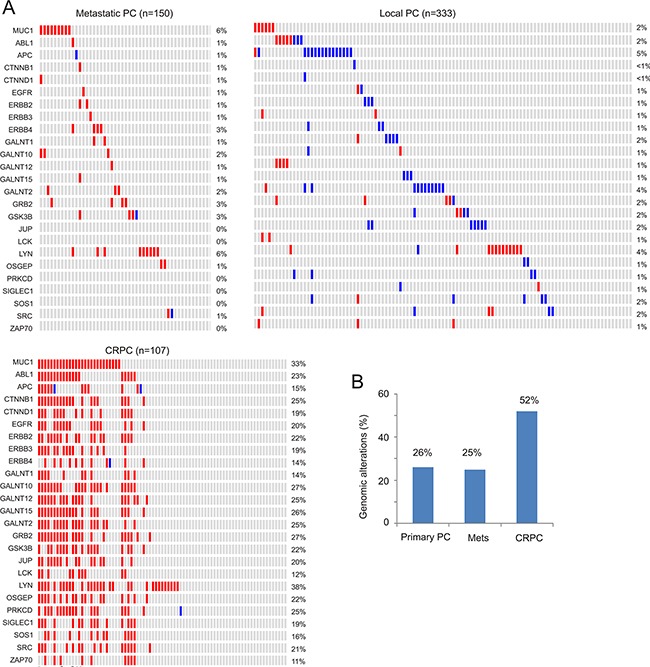
Genomic alterations of genes in the MUC1 network The MUC1 network (see Supplementary Figure S4 and Supplementary Table S2 for details) was analyzed for genomic alterations using the 3 largest and representative prostate cancer datasets within the cBioPortal database [44, 45]. These datasets were deposited from studies as a result of their publications [46–48], and cover 333 primary PCs [46], 150 metastatic PCs [47], and 107 CRPCs derived from 77 patients [48]. **A.** Analyses were performed using the tools provided by cBioPortal [44, 45]. For the primary and metastatic population, only a proportion of cohorts containing the tumors with the relevant genomic alterations are included. Each column represents an individual tumor; red and blue slots are for gene amplification and deep deletion, respectively. Gene names and their rates of alteration are shown on the left and right of individual rows, respectively. **B.** Summary of genomic alterations in the MUC1 network in the primary, metastatic and castration resistant PC cohort.

To further study the relationship between MUC1 gene amplification and CRPC, we noticed a MUC1 gene network consisting of the seed node MUC1 and 24 linker nodes, which were generated using the cBioPortal system (see Supplementary Figure S4 for details). The network contains two major components (see Discussion for a second major group): notably, a group of tyrosine kinases including EGFR and non-receptor tyrosine kinases (ABL1, ERBB2-4, LCK, LYN, SRC1, and ZAP70) (Supplementary Figure S4). These proteins have been demonstrated to have functional connections with MUC1-C [16, 17] and contribute to PC and CRPC development (Supplementary Table S2). In accordance with this knowledge, our analyses revealed that genomic alteration (amplification or deep deletion) of these genes occurred in 26% of primary PCs, 25% of metastatic PCs, and 52% of CRPCs (Figure 7).

**Figure 8:**
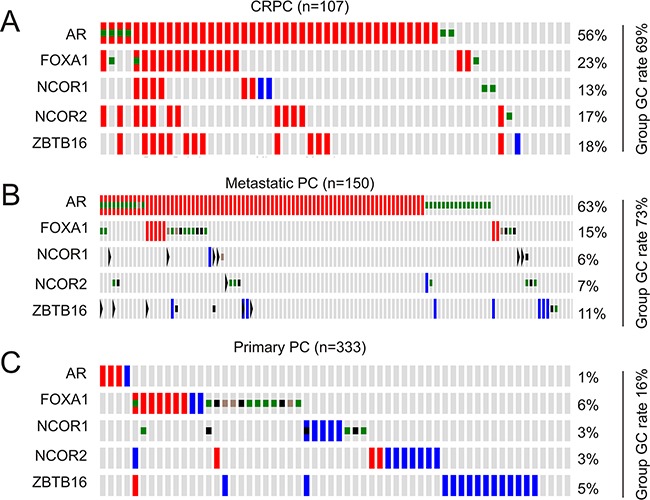
Genomic changes in the AR gene and its coregulators Genomic alterations in the AR gene, FOXA1, NCOR1, NCOR2, and ZBTB1 were analyzed in the primary, metastatic, and castration resistant PC cohort within the cBioPortal database [44, 45]. The group rates of genomic change (GC) are provided. Red and blue slots are for amplification and deep deletion, respectively; green, brown, and black squares indicate missense, inframe, and truncating mutations, respectively.

To further examine association of the MUC1 network with CRPC development, we investigated it in the context of AR signalling. Persistent AR signalling by alterations in AR, a pioneer factor FOXA1, and three steroid receptor coactivators (NCOA1/SRC-1, NCOA2/SRC-2, and NCOA3/SRC-3) is known to contribute to CRPC progression [5, 49]. NCOA2 activates AR signaling [5, 42]; its amplification occurs in 24.3% of metastatic PCs. Although evidence supports that AR transcriptional activity is repressed by nuclear receptor corepressor 1 (NCOR1), NCOR2, and PLZF/ZBTB16 [50–52], genomic alterations in AR, FOXA1, and these corepressors were reported in a large CRPC cohort [48]. We thus analyzed genomic alterations (GNs) in AR and its coactivators (FOXA1 and NCOA1-3) or AR coregulators (FOXA1, NCOR1, NCOR2, and ZBTB16). GNs in the AR gene predominantly occurred in the form of amplification, in 56% of CRPCs (Figure 8A). Consistent with the MUC1 gene network's concurrent GCN increases in individual CRPCs (Figure 7), co-amplification of AR and its coregulators (Figure 8A) or coactivators (Supplementary Figure S5) was also observed. In primary PC, metastatic PC, and CRPC populations, the AR gene was altered in 1%, 63%, and 56% of tumors, respectively (Figure 8). In these cohorts, the MUC1 gene was amplified in 2%, 6%, and 33%, respectively (Figure 7). Genomic alterations occurred in 16%, 73%, and 69% in the above cohorts for the AR coregulator group respectively, (Figure 8) as well as 14%, 73%, and 71% for the AR coactivator group (Supplementary Figure S5). Corresponding changes in the MUC1 network were 26%, 25%, and 52% (Figure 7B). Collectively, these analyses support the concept that genomic alterations in MUC1 and its gene network are as specific as AR and its coregulator group for metastasis and CRPC progression.

### Genomic alterations in the MUC1 network associate with CRPC adenocarcinoma and neuroendocrine PC (NEPC)

**Figure 9:**
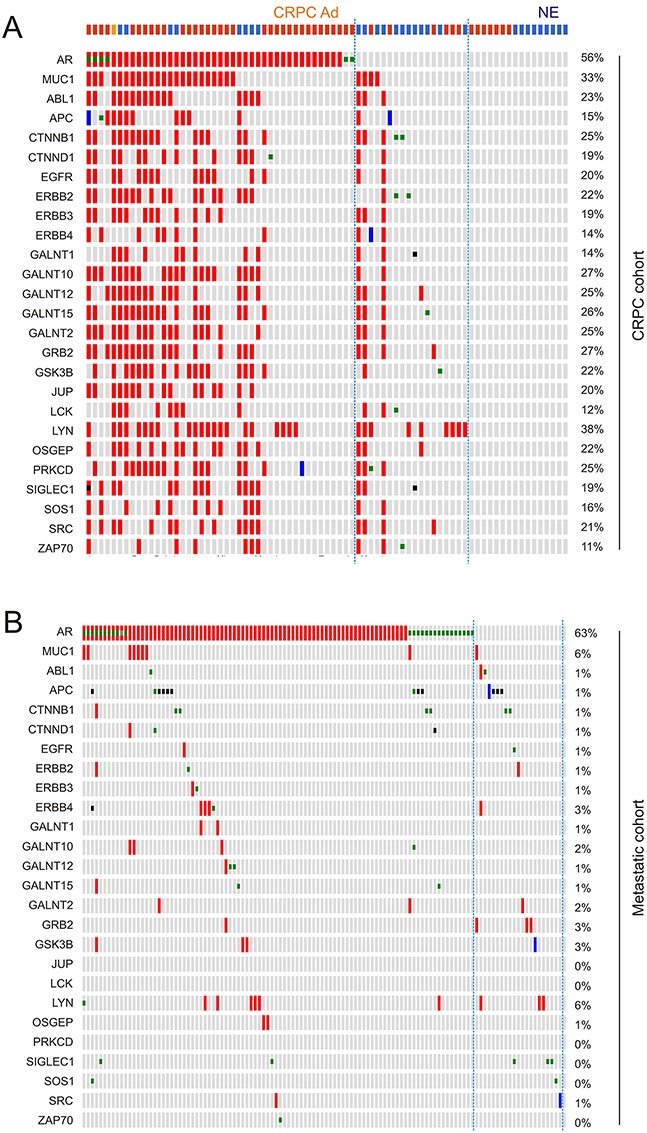
Concurrent and independent genomic alterations between the AR gene and the MUC1 genes network Gene amplification (red slot), deep deletion (blue slot), missense mutation (green square), inframe mutation (brown square), and truncating mutation (black square) in the AR gene and those genes in the MUC1 network were extracted from the CRPC and metastatic PC dataset within cBioPortal [44, 45]. **A.** The top row demonstrates pathologies for individual CRPC: red for CRPC adenocarcinoma (CRPC Ad); blue for neuroendocrine PC (NE); and orange for CRPC adenocarcinoma mixed with NEPC. CRPC cases bordered by two dot lines have genomic changes only in the MUC1 network. **B.** Genomic alterations in the AR gene and the MUC1 network in a metastatic PC cohort.

We subsequently analyzed genomic alterations in the MUC1 network and AR gene. Genomic alterations (amplification, deep deletion, and mutations) in the MUC1 gene network display both concordant and independent signatures with the AR gene (Figure 9A). A large proportion of changes detected in the MUC1 network overlap with those observed in the AR gene (Figure 9A). In CRPC however, a minor proportion of genomic alterations to the MUC1 network occur independently of AR (Figure 9A, see those CRPCs lined with two dot lines). In the latter group, the JUP gene displays no changes (Figure 9A). The most interesting feature in this group is the enrichment of NEPC cases, which compose 66.7% (12/18) of the CRPCs (Figure 9A), suggesting that this gene signature (AR and JUP genes are genomic alteration free, while there are a number of genomic changes in the MUC1 network) shows greater specificity towards this type of CRPC.

While in the primary PC cohort independency was observed between genomic alterations of the MUC1 network and AR gene (Supplementary Figure S6), both concordance and independency could be identified in the metastatic PC cohort (Figure 9B). Since all metastatic PCs will progress to CRPC as either adenocarcinoma or NEPC, it will be interesting to examine whether metastases with genomic alterations only in the MUC1 network will progress into NEPCs.

To further evaluate the association of genomic alterations in the MUC1 network with NEPC, we analyzed an association between loss of RB and NEPC. Among 8 CRPCs with *RB1* gene mutations (6 deep deletions and 2 missense mutations), 6 (6/8=75%) were NEPCs (Supplementary Figure S7). RB loss is a typical event/marker for NEPC [53]. In this regard, the enrichment of NEPCs in CRPCs with genomic alterations in the MUC1 network (12/18=66.7%) is similar to the enrichment associated with *RB1* genomic alterations.

### Genomic alterations in the MUC1 network associate with a reduction in disease free survival (DFS)

CRPC is a major form of PC progression; the association of genomic alterations in the MUC1 gene network with CRPC strongly suggests a correlation of these changes with PC recurrence (DFS) and/or overall survival (OS). To test this possibility, we first performed a time-to-event analysis using a Kaplan-Meier curve to determine whether elevation of MUC1 mRNA associates with rapid kinetics of PC metastatic progression. By using the data available from the Grasso dataset within Oncomine^TM^ (Compendia Bioscience, Ann Arbor, MI), we could not observe a correlation (Supplementary Figure S8).

**Figure 10:**
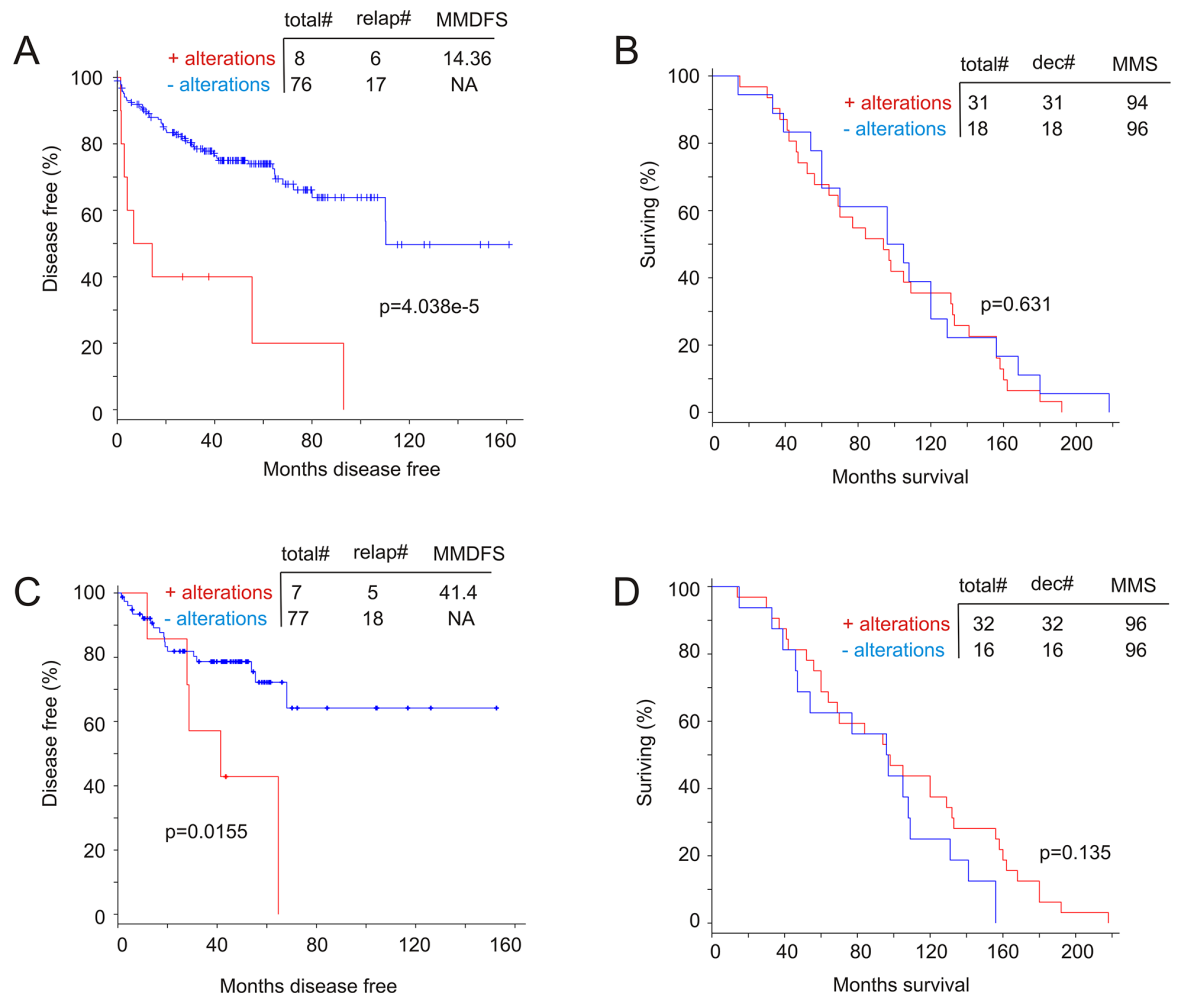
Genomic alterations in the MUC1 network associate with a reduction in disease free survival (DFS) A dataset of primary PC [42] **A, C.** and a dataset of metastatic PC [41] **B, D.** within the cBioPortal database [44, 45] were used to assess the impact of the MUC1 network on DFS (**A**) and OS (**B**) as well as the effects of the AR network on DFS (**C**) and (**D**). Statistical analysis was performed using logrand test. Total#: total number of cases; relap#: number of relapsed cases; dec#: number of deceased cases; MMDFS: median months disease free survival; MMS: median months survival; NA: not available.

We subsequently examined a potential association of genomic alterations in the MUC1 network with DFS and OS. Among the 10 datasets related to genomic alteration in PC from cBioPortal [44, 45], one contained follow-up data for PC relapse of 84 patients [42] and another had follow-up information for PC-related mortality of 49 cases [41]. Consistent with a small number of cases with MUC1 genomic alterations in a large primary PC and metastasis population (Figure 7), MUC1 genomic alterations were also infrequently detected in these small cohorts [41, 42] (Supplementary Figure S9). To perform a meaningful Kaplan-Meier analysis, we thus used cases with and without alterations in the MUC1 network, and are able to show that the genomic alterations significantly associated with a reduction in DFS (Figure 10A) but not OS (Figure 10B). Similar observations were also obtained for the AR coregulator group (Figure 10C, 10D); the detailed genomic alterations for this group are included in Supplementary Figure S10. Surprisingly, genomic changes in the AR coactivator group do not correlate with either DFS or OS (Supplementary Figure S11). Collectively, genomic alterations in the MUC1 network or the AR coregulator group facilitate PC recurrence, but not patient survival following metastatic events. Nonetheless, by comparing the reduction curve and median months disease free survival (MMDFS) associated with genomic alterations in the MUC1 network to those in the AR coregulator group, the former is likely to have a greater impact on promoting PC recurrence.

## DICUSSION

MUC1 is the most attractive TAA, a status that is attributable not only to its dramatic alterations in cancer but also to the prevalence of these changes across multiple tumor types [11, 12, 17]. Furthermore, the MUC1-C subunit promotes the actions of multiple critical oncogenic pathways, including those of EGFR, ERBB, non-receptor tyrosine kinases, β-catenin, NF-κB, PKM2, and others [16, 17, 54]. However, although there is evidence supporting an association of MUC1 amplification with PC tumorigenesis, this relationship seems variable depending on the antibodies used. To make this issue even more complex, two short forms of MUC1 (MUC1/Z and MUC1/Y) were reported in 3 benign prostatic hyperplasias (BPH) and 3 primary PC tissues in 2003 [55]. Both MUC1/Z and MUC1/Y are missing the N-terminal tandem repeats, and may retain a short fragment of MUC1-N [38]. However, their function in tumorigenesis remains unclear. Collectively, the relationship between MUC1 expression and PC progression has not been thoroughly investigated or established.

By employing comprehensive experimental systems and analyses involving *in vitro* and *in vivo* studies, as well as examining the changes in MUC1 protein, mRNA, and genomic DNA, our research reveals several novel observations: MUC1 upregulation associates with PCSLCs, MUC1 levels are increased in the late phases of PC progression (metastasis and CRPC development), there is specific copy number amplification of the MUC1 gene and its associated gene network in CRPC, genomic alterations in the MUC1 network together with non-alteration of the AR gene could be potentially utilized as an NEPC signature, and the correlation of genomic alterations in the MUC1 network with PC relapse.

For the association with PC recurrence, it appears that not all MUC1 network associated genes display genomic alterations in the primary PC cohort (Supplementary Figure S9, left panel). As expected, removal of these unchanged genes does not affect the network's association with DFS based on this cohort (data not shown). While this may suggest that the remaining 12 genes (ABL1, APC, CTNNB1, EGFR, ERBB2, ERBB3, ERBB4, GALNT1, GALNT2, GRB2, LYN, and SOS1) are a signature of PC relapse, it should be taken with caution as this cohort is rather small. Future research will need to address this issue.

Our observed upregulation of MUC1 in PCSLCs suggests a mechanism underlying MUC1 overexpression in PC. It is becoming clearer that the plasticity of cancer stem cells is critical in cancer evolution in response to endogenous and exogenous selective pressures (such as therapies). This plasticity likely contributes to the acquisition of new properties that drive cancer metastasis and development of therapeutic resistance. In this regard, PCSC-associated plasticity may contribute to MUC1 upregulation. In support of this possibility, we have recently reported a new PC metastatic factor contactin 1 (CNTN1) that was also derived from PCSLCs [56]. In a similar manner, PCSC-derived MUC1 may be specifically important for metastatic progression, considering genomic amplification in the MUC1 gene is an infrequent event in metastatic PCs (Figure 7).

In CRPC however, genomic amplification is likely a major mechanism underlying MUC1 upregulation (Figure 7). It is likely that androgen deprivation is a contributing factor to MUC1 gene amplification in CRPC. This concept is supported by the importance of persistent AR signalling in CRPC development [5] and in promoting genomic instability [57–59]. Additionally, this concept is also in line with the observed concordance between AR gene amplification and MUC1 GCN increases in CRPC (Figure 9A). However, the genomic amplifications which occur in MUC1 and its gene network are not merely a side effect of AR signalling-caused genomic instability, which can be reflected by the independent genomic alterations between the MUC1 network (Figure 7) and the AR groups (Figure 8, Supplementary Figure S5).

The MUC1 network contains 9 tyrosine kinases (ALB1, EGFR, ERBB2, ERBB2, ERBB4, LCK, LYN, SRC1, and ZAP70), GRB2, and SOS1 (Supplementary Table S2); both GRB2, and SOS1 facilitate tyrosine kinase signalling [60]. Thus, proteins contributing to tyrosine kinase function compose 45.8% (11/24) of the network's linker nodes (Supplementary Figure S4). Tyrosine kinase activity is well known to promote cancer progression, which is likely a major attribute to the specific association of the MUC1 network and CRPC. The second major group of proteins in the MUC1 network are enzymes (GALNT1, GALNT2, GALNT10, GALNT12, GALNT15, OSGEP, and SIGLEC1) that contribute to MUC1 glycosylation (Supplementary Table S2). The involvement of these proteins in tumorigenesis has not been well studied. Nonetheless, their co-amplification with MUC1 in CRPC suggests a contribution to at least the generation of MUC1 as a CRPC-associated antigen. The MUC1 network also possesses APC1, JUP, and PRKCD genes that display tumor suppression functions (Supplementary Table S2), which are also co-amplified (Supplementary Table S2). The function of their amplification remains unclear. It may be a result from the prevalence of genomic amplification over deletion in the CRPC cohort [48]. However, it will be a challenge to reconcile their amplification in CRPC with their deep deletion in primary tumors (Figure 7). The same situation also applies to GALNT2 (Figure 7). It is a provocative thought that the primary PCs with these deletions are unlikely to progress to CRPC. An equally provocative possibility is that their amplification contributes to CRPC development in some way. These uncertainties may be solved in the future. Nonetheless, our analysis supports an intriguing connection of the MUC1 network with CRPC.

Similar ambiguities are also observed in the AR groups. While co-amplification of the AR gene with its coactivators is in line with the importance of persistent AR signalling in CRPC development [5], co-amplification of AR with its repressors (NCOR1, NCOR2, and ZBTB16) may play a different role in the process. Both NCOR1 and NCOR2 reduce agonist and antagonist-elicited AR activity [61], indicating a complex role of these factors in modulating AR function. Intriguingly, addition of NCOR1, NCOR2, and ZBTB16 to AR and FOXA1 empowers an association with PC recurrence (Figure 10C); in fact AR plus NCOR1, NCOR2, and ZBTB16 is almost sufficient to predict a reduction in DFS (Supplementary Figure S12). Intriguingly, the AR coactivator group does not possess this ability (Supplementary Figure S11), suggesting that co-amplification of AR with its repressors impacts CRPC development.

Considering the well-established associations of genomic alterations in the AR and its co-activator genes with CRPC [5, 42, 48, 49, 62], it is confirmative that respective aberrations in the MUC1 network are as specific as those in the AR in identifying CRPC. This concept needs to be further examined. Nonetheless, it is rather intriguing that genomic alterations in the MUC1 network and AR largely overlap (Figure 9A), indicating a positive regulation between AR and MUC1. However, current evidence supports the opposite possibility of mutual inhibition [63, 64]; this negative feedback may contribute to the MUC1 network's unique genomic changes in NEPCs (Figure 9A), suggesting MUC1's contribution to the generation of NEPC during ADT.

In our investigation we've uncovered a specific upregulation of MUC1 in CRPC, which is not only novel but also implies a role of MUC1 in CRPC development. In accordance with the essential contribution of PCSCs in CRPC progression [36], the increase of MUC1 levels in our PCSLCs (Figure 1) provides additional support to this statement. This development would suggest an addition of a MUC1-based immunotherapy during ADT. This combination is particularly appealing in view of our recent phase I/II clinical trial using dendritic cell-based MUC1 vaccination in treating patients with CRPC. Furthermore, a recent publication reported a significant survival advantage to combining docetaxel and ADT, compared to ADT alone, in treating patients with metastatic PC [65]. Based on MUC1's association with PCSLCs and upregulation of MUC1 expression following docetaxel treatment reported here, it could be expected that combinational therapy involving docetaxel, ADT, and MUC1-based immunotherapy may provide an additional survival benefit over that of just docetaxel+ADT. MUC1 has already been explored for cancer immunotherapy strategies based on cancer-associated alterations in MUC1-N. However, a recent development identifying GO-203 that specifically targets MUC1-C [66] instead is an attractive addition to MUC1-based therapies for CRPC.

## MATERIALS AND METHODS

### Cell culture and generation of DU145 spheres (PCSLCs)

LNCaP, PC3, and DU145 cells were purchased from American Type Culture Collection (ATCC), and cultured in RPMI-1640 (LNCaP), F12 (PC3) and MEM (DU145) media supplemented with 10% FBS (Sigma Aldrich) and 1% Penicillin-Streptomycin (Thermo Fisher Scientific). DU145 spheres were generated and cultured according to our published conditions [37]. Briefly, DU145 monolayer cells (non-PCSLCs) were individualized and seeded at a density of 5,000 cells/mL in serum-free (SF) media (3:1 DMEM/F12 mixture) (Thermo Fisher Scientific) containing 0.4% bovine serum albumin (BSA) (Bioshop Canada Inc.) supplemented with 0.2xconcentration of B27 minus Vitamin A (Thermo Fisher Scientific) and 10ng/ml EGF (Sigma Aldrich), in T75 flasks. Typical spheres were formed in 10 to 12 days.

### Collecting primary prostate cancer

Prostate biopsies and radical prostatectomy tissues were obtained at St. Joseph's Hospital in Hamilton, Ontario, Canada under approval from the local Research Ethics Board (REB# 11-3472) and with patient consent.

### Xenograft tumor formation and docetaxel treatment

DU145 monolayer (non-PCSLCs) and sphere (PCSLCs) cells were resuspended in 0.1 ml MEM/Matrigel mixture (BD) (1:1 volume), followed by subcutaneous implantation into the flanks of 8 week-old male NOD/SCID mice (The Jackson Laboratory). 10^6^ DU145 monolayer cells and 10^4^ DU145 sphere cells were implanted, based on our previous report that DU145 spheres display a 100-fold higher capacity to form xenografts [37]. Tumors were assessed through observation and palpation, and tumor growth was measured weekly using calipers. Tumor volume was determined using the formula V = L x W^2^ x 0.52. Once tumors reached a volume of at least 100mm^3^, mice were treated with either DMSO control or docetaxel (Santa Cruz) at 10mg/kg once a week for three weeks by intraperitoneal injection. After a week of recovery, treatment was repeated until tumors reached a volume ≥ 1000 mm^3^, at which point animals were sacrificed. All animal work was carried out according to experimental protocols approved by the McMaster University Animal Research Ethics Board.

### Western blot analysis

Cells were lysed in a buffer containing 20mM Tris (pH 7.4), 150mM NaCl, 1mM EDTA, 1mM EGTA, 1% Triton X-100, 25mM sodium pyrophosphate, 1mM NaF, 1mM β-glycerophosphate, 0.1mM sodium orthovanadate, 1mM PMSF, 2μg/ml leupeptin and 10μg/ml aprotinin. 50μg of whole cell lysate was separated on SDS-PAGE gel, and transferred onto Hybond ECL nitrocellulose membranes (Amersham), followed by blocking with 5% skim milk at room temperature for one hour. Primary antibodies were incubated overnight at 4°C with agitation, and secondary antibodies incubated for one hour at room temperature. Signals were then developed (ECL Western Blotting Kit, Amersham). Primary antibodies: anti-MUC1-N 1:50 (BD) and anti-Actin 1:1000 (Santa Cruz).

### Quantitative real-time PCR analysis of MUC1 expression

Total RNA was isolated with TRIZOL, and reverse transcription was carried out using superscript III (Thermo Fisher Scientific) according to the manufacturer's instructions. Quantitative real-time PCR was performed using the ABI 7500 Fast Real-Time PCR System (Applied Biosystems) using SYBR-green (Thermo Fisher Scientific). All samples were run in triplicate. MUC1 (Forward): 5′-TGCCGCCGAAAGAACTACG-3′, MUC1 (Reverse): 5′-TGGGGTACTCGCTCATAGGAT-3′. β-Actin (Forward): 5′-ACCGAGCGCGGCTACAG-3′, β-Actin (Reverse): 5′- CTTAATGTCACGCACGATTTCC -3′.

### Immunohistochemistry (IHC)

IHC was performed on paraffin embedded and serially cut prostate cancer tissues obtained from St. Joseph's Hospital, Hamilton, Ontario, Canada. Slides were deparaffinized in xylene and cleared in an ethanol series. Antigen retrieval was performed in a food steamer for 20 minutes using sodium citrate buffer (pH = 6.0). Tissues were blocked for 1 hour in PBS containing 1% BSA and 10% normal goat serum (Vector Laboratories). MUC1-N (1:200, BD) and MUC1-C (1:10, Fishr Scientific) antibodies were incubated overnight at 4°C. Secondary antibody biotinylated goat anti-mouse IgG or anti-hamster IgG, respectively, and Vector ABC reagent (Vector Laboratories) were incubated according to the manufacturer's instructions. Secondary antibody only was used as negative control. Washes were performed with PBS. Chromogenic reaction was carried out with diaminobenzidine (Vector Laboratories), and slides were counterstained with haemotoxylin (Sigma Aldrich). Image analysis was performed using ImageScope software (Leica Microsystems Inc.). Staining intensity values derived from ImageScope were converted to an HScore using the formula [HScore = (% Positive) x (intensity) + 1]. The HScore was normalized through background subtraction and averaged amongst ≤ 5 images per tissue sample.

### Statistical analysis

Statistical analysis was performed using student t-test, with p < 0.05 being considered statistically significant.

## SUPPLEMENTARY FIGURES AND TABLES




