# Supplementary material for: Amplification of MUC1 in prostate cancer metastasis and CRPC development
Source: Oncotarget. 2016 Nov 4;7(50):83115–33. doi: 10.18632/oncotarget.13073 (PMC5347757; doi:10.18632/oncotarget.13073)
Supplement: Supplementary file 1 [file oncotarget-07-83115-s001.pdf]

Amplification of MUC1 in prostate cancer metastasis and CRPC development

SUPPLEMENTARY FIGURES AND TABLES

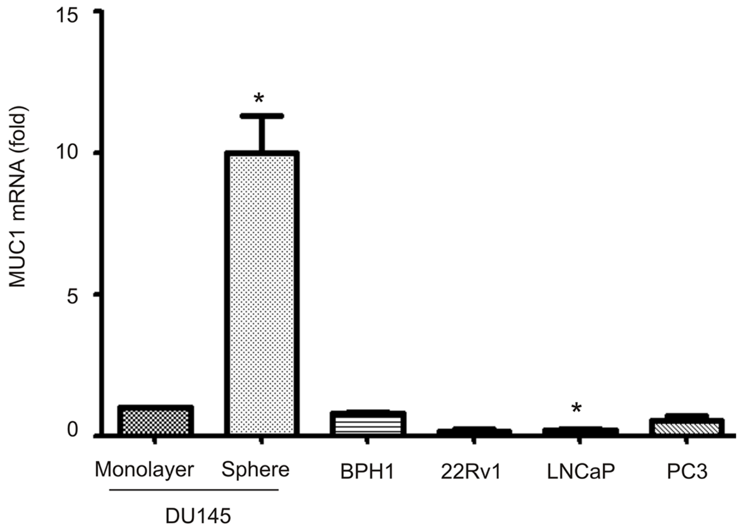

Supplementary Figure S1: Elevation of MUC1 mRNA expression in PCSLCs. Real time PCR analysis of MUC1 mRNA in DU145 monolayer, DU145 sphere (PCSLCs), BPH1 (immortalized human prostate epithelial cells), 22Rv1, LNCaP, and PC3 cells. Experiments were repeated three times; means±SE are graphed. *$p<0.05$ by a 2-tailed Student's t-test in comparison to DU145 monolayer cells; $p=0.0525$ for 22Rv1 cells.

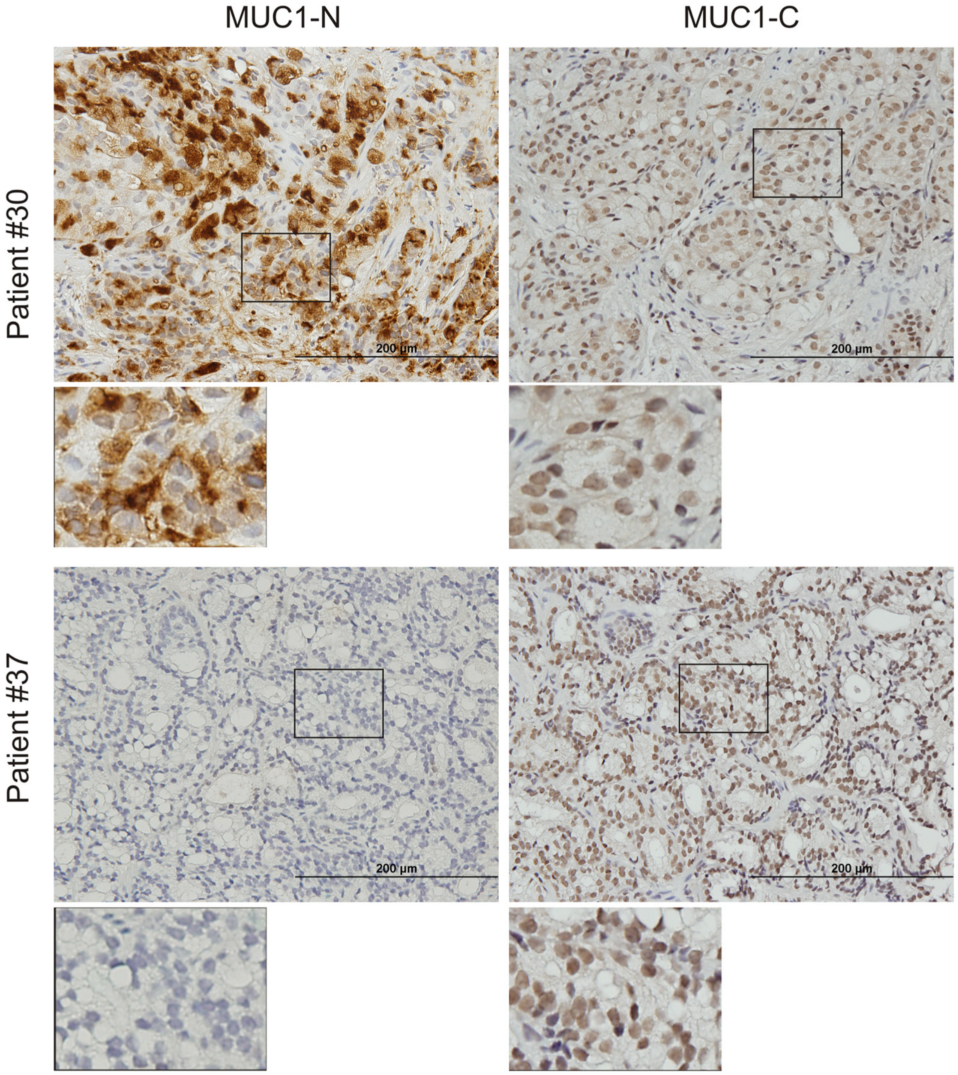

Supplementary Figure S2: Examination of MUC1 protein expression in primary PC using anit-MUC1-N (MUC1-N) or anti-MUC1-C (MUC1-C) antibody. The individual patients examined are shown. The indicated areas are enlarged 2.2 fold and placed under the respective panels.

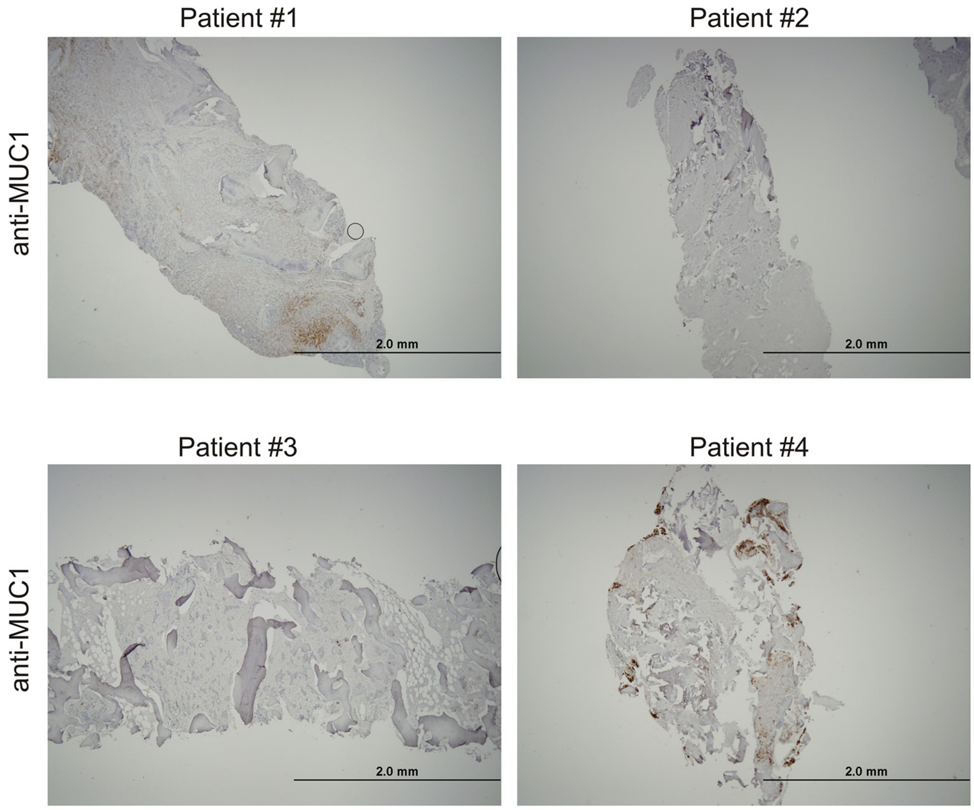

Supplementary Figure S3: The overall IHC staining for MUC1 in four bone PC metastases. Scale bar represents 2mm.

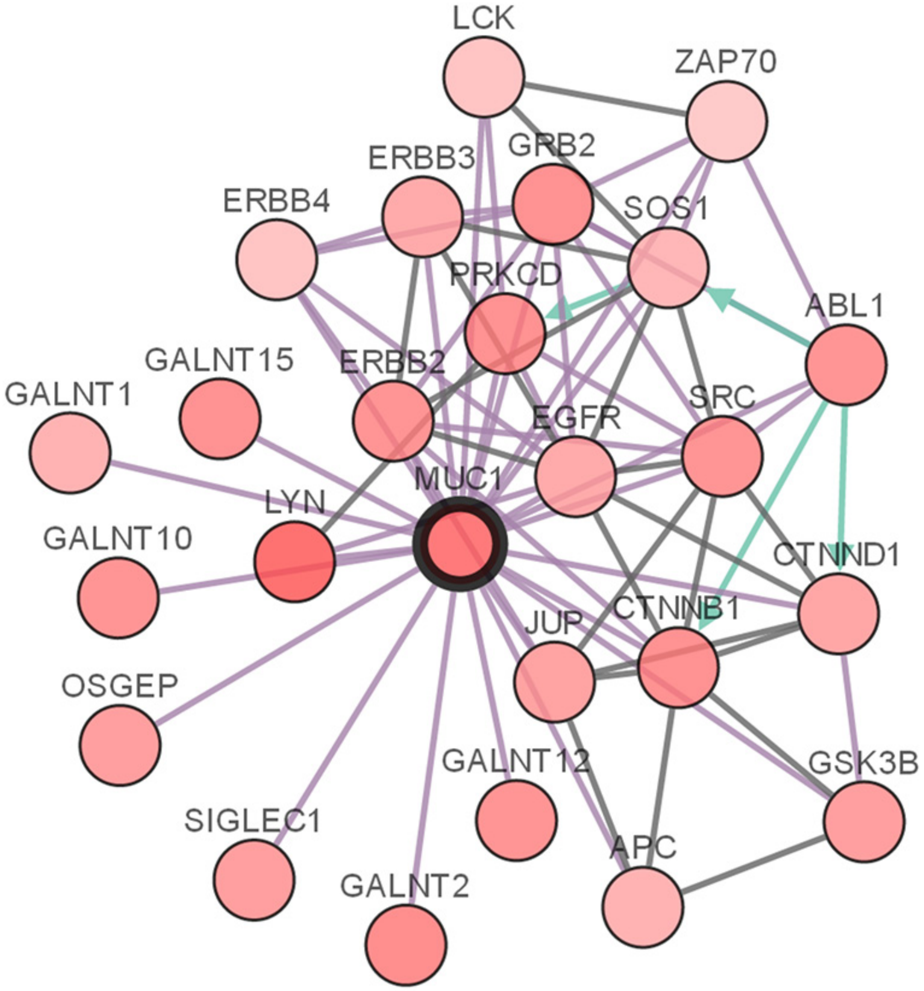

Supplementary Figure S4: The MUC1 network. The network was generated using MUC1 as the seed node; the liner nodes were identified using the pathway data and interaction data from HPRD, Reactome, NCI-Nature Pathway Interaction Database, and the MSKCC Cancer Cell Map. The pathway was constructed by the system provided by cBioPortal (http://www.cbioportal.org/) (also see Supplementary Table S2).

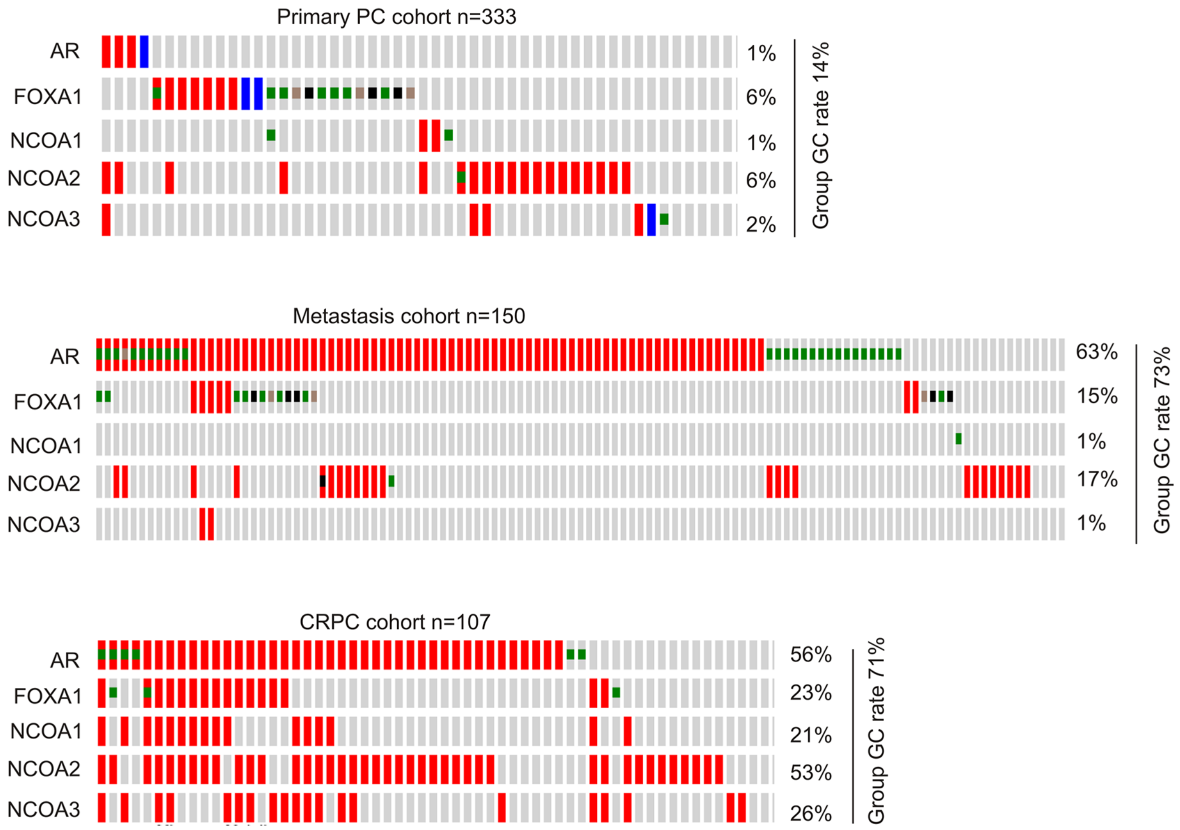

Supplementary Figure S5: Genomic alterations of genes in the AR coactivator group. The AR, FOXA1, NCOA1, NCOA2, and NCOA3 genetic alterations in the indicated datasets were analyzed. These datasets were deposited from the respective publications [46-48] to cBioPortal. For each database, only the proportions of tumors containing the indicated genomic changes are included. Each column represents an individual tumor. Gene names and their rates of alteration are shown on the left and right of individual rows, respectively. The group rates of genomic changes (GC) are also provided. Red and blue slots are for gene amplification and deep deletion, respectively; green, brown, and black squares represent missense, inframe, and truncating mutations, respectively.

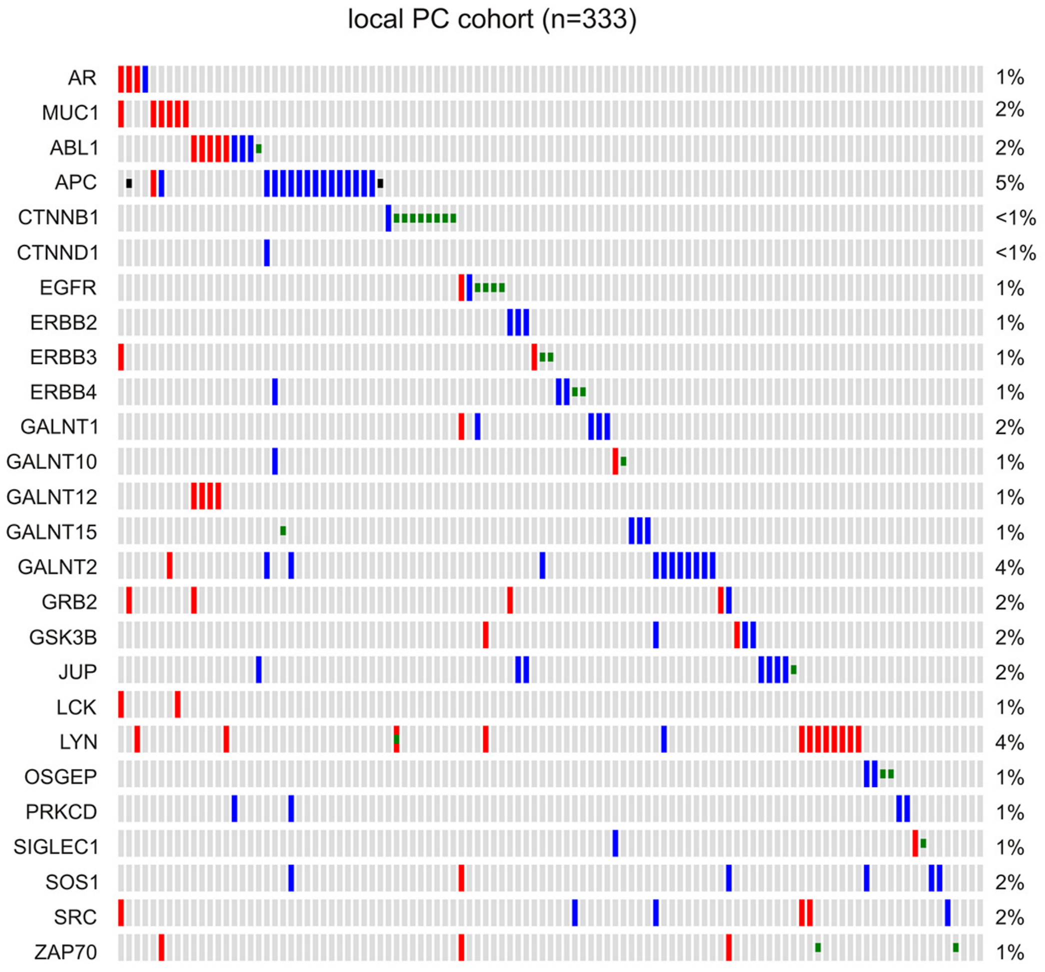

Supplementary Figure S6: Independent occurrence of the genomic alterations in the AR gene and the MUC1 network. Gene amplification (red slot), deep deletion (blue slot), missense mutation (green square), and truncating mutation (black square) in the AR gene and the MUC1 network genes were extracted from the largest cohort of primary PC within cBioPortal. Genomic alterations in the AR gene and the MUC1 network in the cohort are shown.

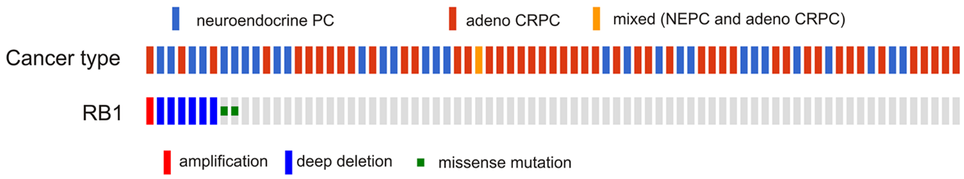

Supplementary Figure S7: Loss of RB is enriched in neuroendocrine PC. Analysis was performed using a dataset [48] within cBioPortal. Adeno: adenocarcinoma.

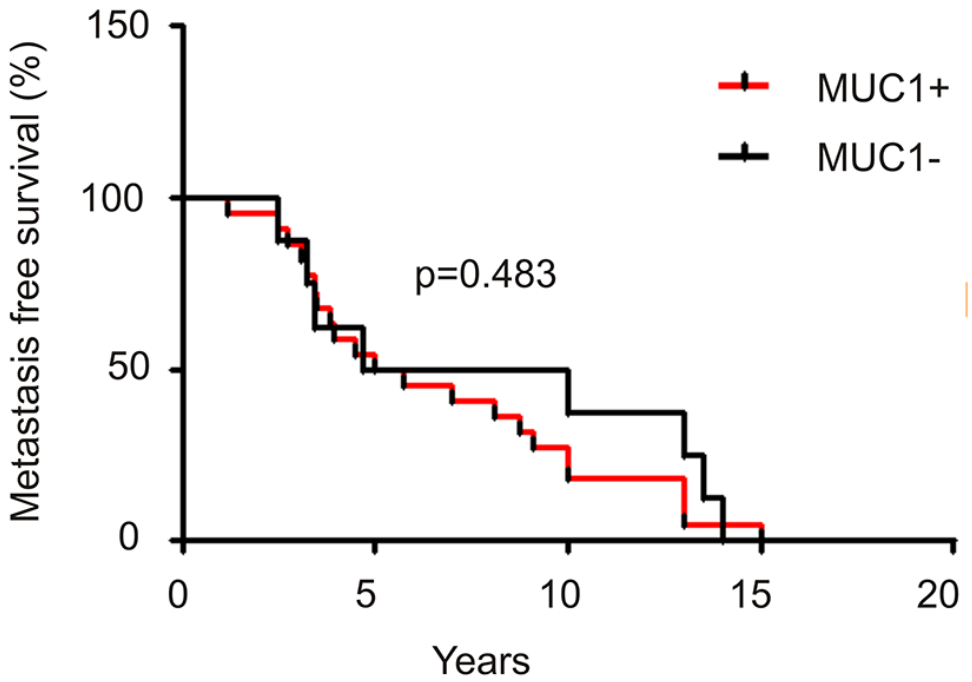

Supplementary Figure S8: High levels of MUC1 mRNA do not enhance PC metastasis. Data was extracted from the Grasso dataset within OncomineTM and analyzed for metastasis free survival according to MUC1+ (with MUC1 mRNA upregulation) and MUC1- tumors. Statistical analysis was performed using logrand test.

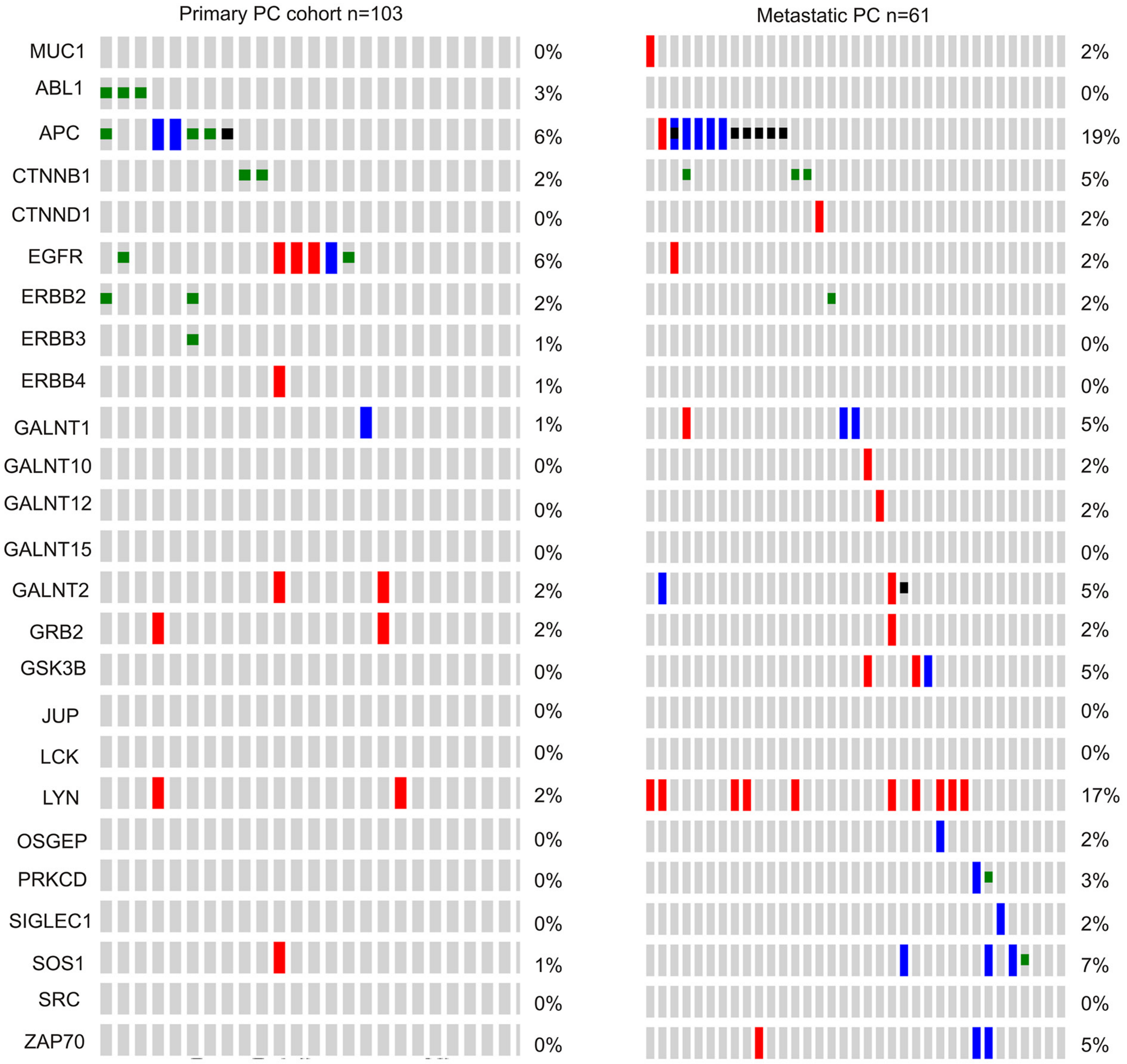

Supplementary Figure S9: Genomic alterations of the MUC1 network in patient cohorts. Genomic data of a primary PC [42] and metastatic PC cohort [41] within cBioPortal were used for the analyses. For each dataset, only the proportions of tumors containing the indicated genomic changes are included. Each column represents an individual tumor. Gene names and their rates of alteration are shown on the left and right of individual rows, respectively. Red and blue slots are for gene amplification and deep deletion, respectively; green and black squares represent missense and truncating mutations, respectively.

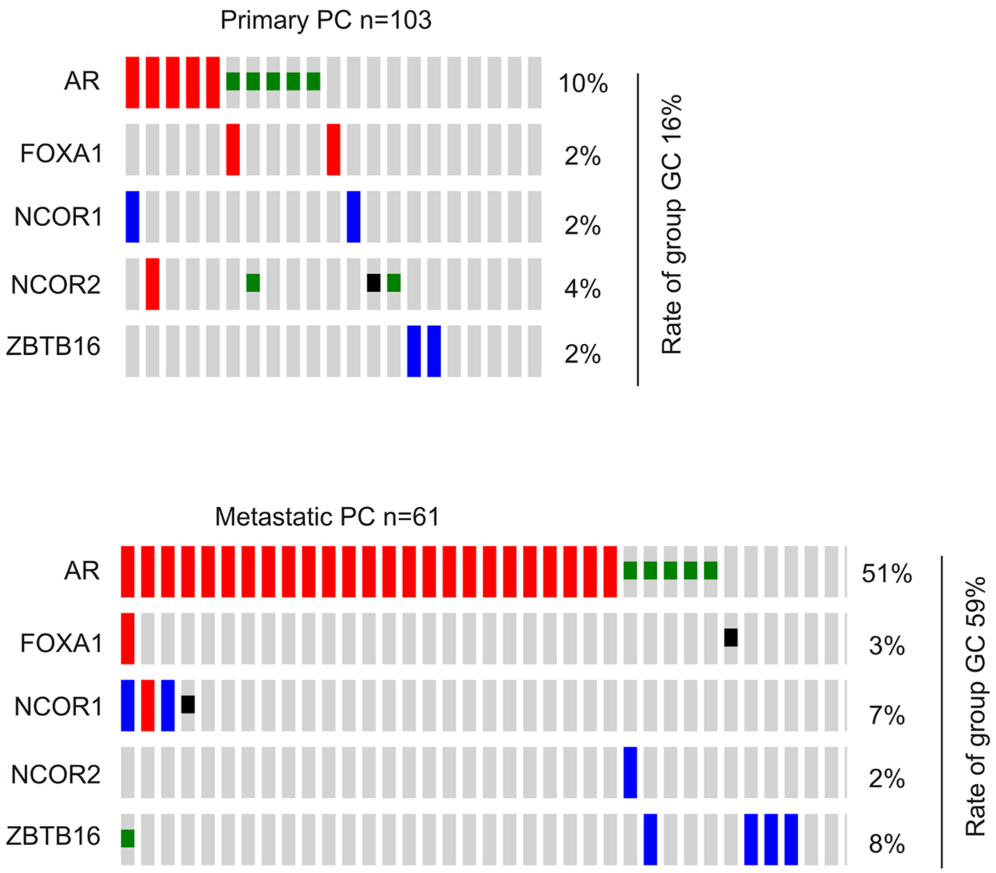

Supplementary Figure S10: Genomic changes in the AR coregulator group. Genomic data of a primary PC [42] **A.** and metastatic PC cohort [41] **B.** within cBioPortal were used for the analyses. Gene names and their rates of alteration are shown on the left and right of individual rows, respectively. The group rates of genomic changes (GC) are also provided. Red and blue slots are for gene amplification and deep deletion, respectively; green and black squares represent missense and truncating mutations, respectively.

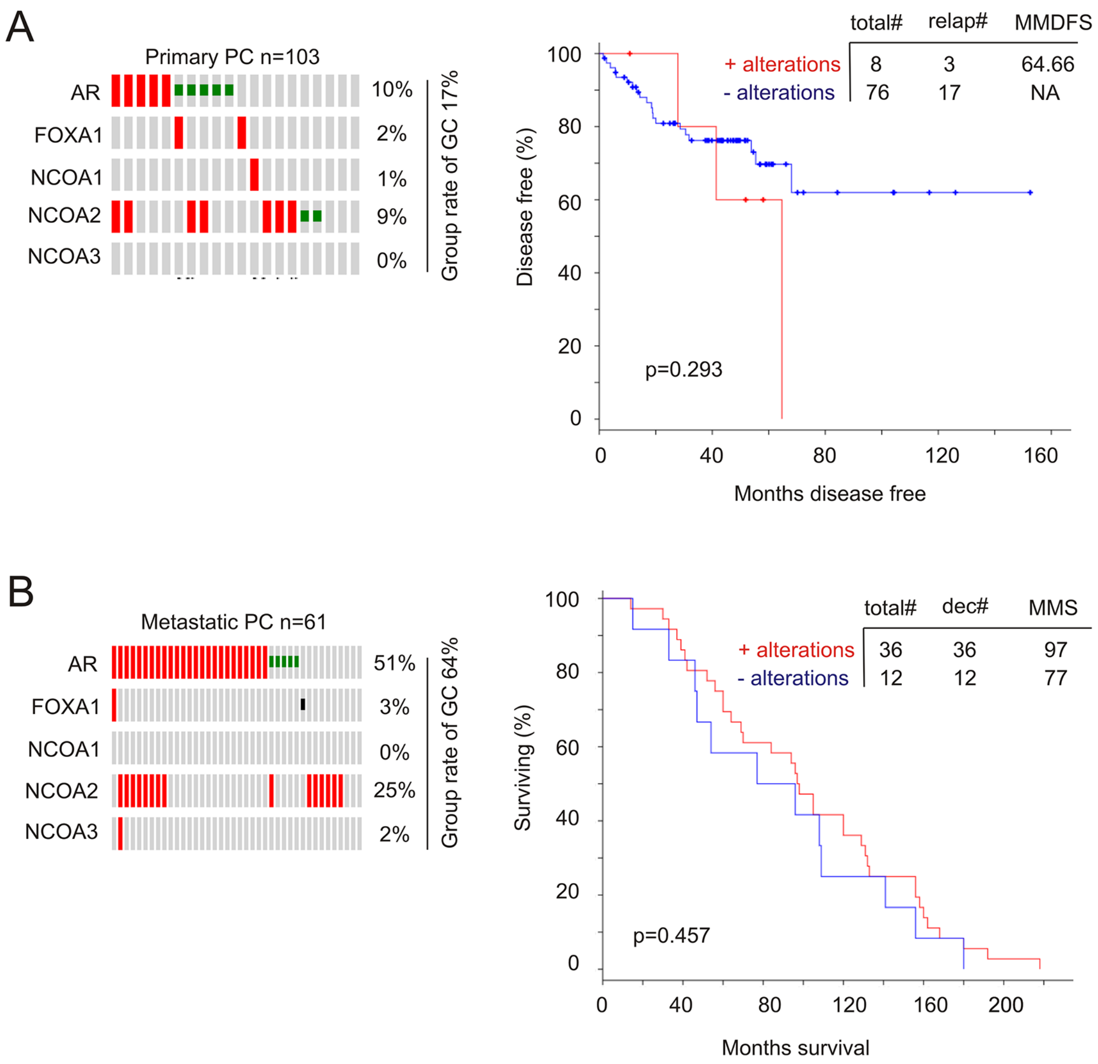

Supplementary Figure S11: Genomic alterations in the AR coactivator group do not correlate with DFS and OS. Primary PC dataset [42] and metastatic PC dataset [41] within the cBioPortal were used to assess the impact on DFS **A.** and OS **B.** Statistical analysis was performed using logrand test. Total#: total number of cases; relap#: number of relapsed cases; dec#: number of deceased cases; MMDFS: median months disease free survival; MMS: median months survival; NA: not available. The respective genomic changes are indicated in the left panels. For each dataset, only the proportions of tumors containing the indicated genomic changes are included; the total number of cases (n) are included. The group rates of genomic changes (GC) are also provided. Red slot: genomic amplification; green square: missense mutation; black square: truncating mutation.

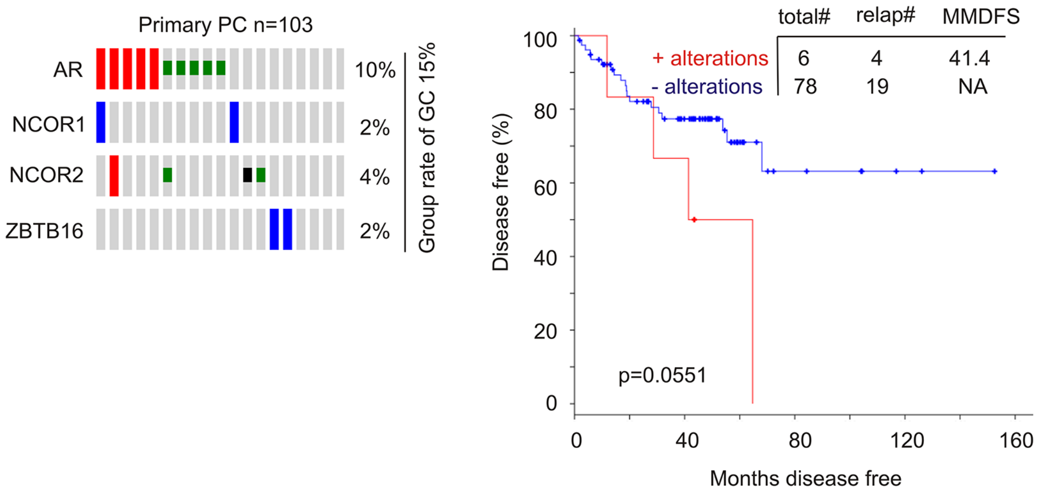

Supplementary Figure S12: Genomic changes in the AR, NCOR1, NCOR2, and ZBTB16 genes are likely associated with a reduction in DFS. A dataset of primary PC [42] within cBioPortal was used to analyze for genetic alterations in these genes (left panel) and for a potential association of these changes with DFS (right panel). Only the proportions of tumors in the cohort containing the indicated genomic changes are included and the total number of cases (n) are shown (left panel). The group rate of genomic changes (GC) is also shown. Red and blue slots are for gene amplification and deep deletion, respectively; green and black squares represent missense and truncating mutations, respectively. Total#: total number of cases; relap#: number of relapsed cases; and MMDFS: median months disease free survival.

Supplementary Table S1: Examination of MUC1 expression using both anti-MUC1-N and MUC1-C antibodies

Patient #[a]	Age	Gleason Score	anti-MUC1-N	anti-MUC1-C
1	76	3+3	++	++
2	55	3+3	++	++
3	53	3+3	++	+++
5	58	3+3	+++	+++
6	59	3+3	++	++
8	-	3+4	+++	+++
12	50	4+3	++	++
14	79	4+4	+	++
18	75	4+5	+	+
22	49	4+5	+++	+++
24	71	4+5	+	+++
26	81	4+5	++	+++
30	80	5+4	+++	+++
34	98	5+5	+++	+++
35	75	3+4	+	++
36	79	3+4	++	+++
37	85	4+4	+	+++
38	69	4+4	+++	+++
39	67	4+4	+++	+++
40	78	5+3	+++	+++

a: 1-34 are the same patients as those in Table 1; patients 35-40 are new patients
+ weak positive, ++ positive, +++ strong positive

Supplementary Table S2: The MUC1 network and its roles in PC progression

See Supplementary File 1
