# Supplementary material for: Amplification of MUC1 in prostate cancer metastasis and CRPC development
Source: Oncotarget. 2016 Nov 4;7(50):83115–33. doi: 10.18632/oncotarget.13073 (PMC5347757; doi:10.18632/oncotarget.13073)
Supplement: Supplementary file 2 [file oncotarget-07-83115-s002.docx]

**Supplementary Table 2**. The MUC1 network and its roles in PC progression

| ***Gene*** | ***Amplification*** | ***Role in PC and CRPC*** | ***Reference*** |
| --- | --- | --- | --- |
| **ABL1** | 20.2% | Abelson tyrosine-protein kinase 1.  Promotes the progression of prostate cancer through activation of proliferation, invasion, tumourigenesis and metastasis. Increased expression observed in prostate cancer tissues. | 1,2 |
| **APC** | 8.8% | Adenomatous polyposis coli. Functions as a scaffold that targets β-catenin for phosphorylation and subsequent proteasomal degradation. In the presence of WNT ligands or APC mutation, β-catenin is no longer degraded and can accumulate in both the cytosol and nucleus where it activates target genes. Allelic deletion of APCs locus in many human cancers suggests it as a tumour suppressor. In PC cases β-catenin is found in the nucleus, suggesting the pathway is frequently de-regulated. This observation corresponds with a decrease in APC mRNA expression. | 3,4 |
| **CTNNB1** | 19.3% | Catenin beta 1. Involved in cadherin-mediated cell adhesion and is under regulation of the Wnt signaling pathway. It localizes to the nuclei in many CRPC tissues and may be involved with advanced PC pathogenesis. | 5 |
| **CTNND1** | 14.9% | Catenin delta 1. Increased expression is correlated with higher PC Gleason score. Overexpression in PC cell lines reduced cell-cell adhesion (E-cadherin) which is seen in PC progression. | 6 |
| **EGFR** | 14.9% | Epidermal growth factor receptor. Increased expression in PC, and is associated with high Gleason and advanced disease (CRPC). When activated, downstream signaling such as AKT and MAPK pathways leads to enhanced proliferation, migration and survival. | 7 |
| **ERBB2** | 18.4% | Erb-B2 receptor tyrosine kinase 2. Overexpression of ERBB2 in androgen independent cells DU145 and PC3 increased metastatic potential in *in vitro* assays. This effect was not seen in androgen dependent LNCaP or Myc-CaP (murine) cells, suggesting a collaboration with androgen signaling. | 8 |
| **ERBB3** | 14.9% | Erb-B2 receptor tyrosine kinase 3. Co-expressed with the AKT, MAPK, and JAK/STAT pathways. Contributes to progression of castration-resistant prostate cancer. While typically expressed in cytoplasm, increased nuclear localization was observed in higher Gleason score and CRPC tissues. | 9,10 |
| **ERBB4** | 8.8% | Erb-B2 receptor tyrosine kinase 4. Approximately 30% of prostate cancer patients present with ERBB4 overexpression. High levels in PC cell lines correlated with greater proliferation rates. | 11,12 |
| **GALNT1** | 12.3% | Polypeptide N-acetylgalactosaminyltransferase 1. Catalyzes the initial reaction in O-linked oligosaccharide biosynthesis, the transfer of an N-acetyl-D-galactosamine residue to a serine or threonine residue on the protein receptor. Has a broad spectrum of substrates for peptides such as EA2, Muc5AC, Muc1a, Muc1b and Muc7. | Uniprot^*,26^ |
| **GALNT2** | 21.9% | Polypeptide N-acetylgalactosaminyltransferase 2. Catalyzes the initial reaction in O-linked oligosaccharide biosynthesis, the transfer of an N-acetyl-D-galactosamine residue to a serine or threonine residue on the protein receptor. Has a broad spectrum of substrates for peptides such as EA2, Muc5AC, Muc1a, Muc1b. Probably involved in O-linked glycosylation of the immunoglobulin A1 (IgA1) hinge region. | Uniprot |
| **GALNT10** | 20.2% | Polypeptide N-acetylgalactosaminyltransferase 10. Catalyzes the initial reaction in O-linked oligosaccharide biosynthesis, the transfer of an N-acetyl-D-galactosamine residue to a serine or threonine residue on the protein receptor. Has activity toward Muc5Ac and EA2 peptide substrates. | Uniprot |
| **GALNT12** | 20.2% | Polypeptide N-acetylgalactosaminyltransferase 12. Catalyzes the initial reaction in O-linked oligosaccharide biosynthesis, the transfer of an N-acetyl-D-galactosamine residue to a serine or threonine residue on the protein receptor. Has activity toward non-glycosylated peptides such as Muc5AC, Muc1a and EA2, and no detectable activity with Muc2 and Muc7. Displays enzymatic activity toward the Gal-NAc-Muc5AC glycopeptide, but no detectable activity to mono-GalNAc-glycosylated Muc1a, Muc2, Muc7 and EA2. May play an important role in the initial step of mucin-type oligosaccharide biosynthesis in digestive organs. | Uniprot |
| **GALNT15** | 20.2% | Polypeptide N-acetylgalactosaminyltransferase 15. Catalyzes the initial reaction in O-linked oligosaccharide biosynthesis, the transfer of an N-acetyl-D-galactosamine residue to a serine or threonine residue on the protein receptor. Although it displays a much weaker activity toward all substrates tested compared to GALNT2, it is able to transfer up to seven GalNAc residues to the Muc5AC peptide, suggesting that it can fill vicinal Thr/Ser residues in cooperation with other GALNT proteins. Prefers Muc1a as substrate. | Uniprot |
| **GRB2** | 21.1% | Growth factor receptor bound protein 2. Indirectly targets androgen receptor through the MAPK pathway, promoting cell proliferation and survival. | 13 |
| **GSK3β** | 16.7% | Glycogen synthase kinase 3 beta. While GSK-3 is upregulated in many cancers, GSK-3β was found to correlate more with higher Gleason score (GSK-3α correlated more with lower Gleason). GSK-3β promoted AR transcriptional activity and AKT activation in 22Rv1 cells. | 14 |
| **JUP** | 16.7% | Junction plakoglobin. An important component of desmosomes and adherence junctions. Studies demonstrate a key role in controlling epithelial cell motility, with low levels leading to higher metastatic potential. In PC cell lines, JUP was found to interact with SOX4 by promoting its nuclear export. In this way, SOX4s transcriptional role in differentiation, proliferation, and cancer progression may be inhibited, suggesting JUP as a tumour suppressor. | 15,16 |
| **LCK** | 8.8% | Lymphocyte cell-specific protein-tyrosine kinase. Aberrantly expressed in prostate cancer tissues with preferential expression in metastatic lesions. May contribute to neoplastic transformation. | 17 |
| **LYN** | 31.6% | Lck/Yes-related novel protein tyrosine kinase. Expressed in PC cell lines, normal prostate epithelia and the majority of PC tissues, it is involved in cell proliferation and anti-apoptosis. Upregulated expression in CRPC. Overexpression increased AR transcriptional activity both *in vitro* and *in vivo*. | 18,19 |
| **OSGEP** | 17.5% | O-sialoglycoprotein endopeptidase. Component of the EKC/KEOPS complex that is required for the formation of a threonylcarbamoyl group on adenosine at position 37 (t_6_A37) in tRNAs that read codons beginning with adenine. The complex is probably involved in the transfer of the threonylcarbamoyl moiety of threonylcarbamoyl-AMP (TC-AMP) to the N6 group of A37. OSGEP likely plays a direct catalytic role in this reaction, but requires other protein(s) of the complex to fulfill this activity. | Uniprot |
| **PRKCD** | 19.3% | Protein kinase C delta. Involved in apoptosis in response to anti-cancer agents, its expression is decreased in PC cells. Overexpression in LNCaP cells reduced cell number by acting as a mediator for phorbol esters. | 20,21 |
| **SIGLEC1** | 16.7% | Sialic acid binding Ig like lectin 1. Acts as an endocytic receptor mediating clathrin dependent endocytosis. Macrophage-restricted adhesion molecule that mediates sialic-acid dependent binding to lymphocytes, including granulocytes, monocytes, natural killer cells, B-cells and CD8 T-cells. Preferentially binds to alpha-2,3-linked sialic acid (By similarity). Binds to SPN/CD43 on T-cells (By similarity). May play a role in hemopoiesis. | Uniprot |
| **SOS1** | 12.3% | Son of sevenless homolog 1. Found to be overexpressed in primary PC cells derived from African American men, who have an increased risk of developing PC. Expression correlated with Gleason score. Knockdown in PC3 and DU145 cells decreased proliferation, migration, and invasion. | 22 |
| **SRC1** | 20.2% | SRC proto-oncogene. Involved in androgen-independent growth, and plays an important role in normal and dysregulated bone functioning (bone metastasis). In local androgen-dependent tumours, expression correlates with more advanced disease. | 23,24 |
| **ZAP70** | 7.9% | Zeta chain of T cell receptor associated protein kinase 70kDa. Overexpressed in PC cell lines and tissues, and promotes migration and invasion. | 25 |

* Protein description taken from The UniProt Consortium (http://www.uniprot.org/)

**References**

1. Ganguly SS, Plattner R. Activation of abl family kinases in solid tumors. Genes Cancer. 2012; 3(5-6): 414-425.
2. Arora S, Saini S, Fukuhara S, Majid S, Shahryari V, Yamamura S, Chiyomaru T, Deng G, Tanaka Y, Dahiya R. MicroRNA-4723 inhibits prostate cancer growth through inactivation of the Abelson family of nonreceptor protein tyrosine kinases. PLoS One. 2013; 8(11): e78023.
3. Bjerke GA, Pietrzak K, Melhuish TA, Frierson HF, Paschal BM, Wotton D. Prostate cancer induced by loss of Apc is restrained by TGFβ signaling. PLoS One. 2014; 9(3): e92800.
4. Gao X, Zacharek A, Grignon D, Liu H, Sakr W, Porter A, Chen Y, Honn K. High-frequency of loss of expression and allelic deletion of the apc and mcc genes in human prostate-cancer. International Journal of Oncology. 1995; 6(1): 111-117.
5. Wan X, Liu J, Lu JF, Tzelepi V, Yang J, Starbuck MW, Diao L, Wang J, Efstathiou E, Vazquez ES, Troncoso P, Maity SN, Navone NM. Activation of β-catenin signaling in androgen receptor-negative prostate cancer cells. Clinical Cancer Research. 2012; 18(3): 726-736.
6. Lu Q, Dobbs LJ, Gregory CW, Lanford GW, Revelo MP, Shappell S, Chen YH. Increased expression of delta-catenin/neural plakophilin-related armadillo protein is associated with the down-regulation and redistribution of E-cadherin and p120ctn in human prostate cancer. Human Pathology. 2005; 36(10): 1037-1048.
7. de Muga S, Hernández S, Agell L, Salido M, Juanpere N, Lorenzo M, Lorente JA, Serrano S, Lloreta J. Molecular alterations of EGFR and PTEN in prostate cancer: association with high-grade and advanced-stage carcinomas. Modern Pathology. 2010; 23(5): 703-712.
8. Tome-Garcia J, Li D, Ghazaryan S, Shu L, Wu L. ERBB2 increases metastatic potentials specifically in androgen-insensitive prostate cancer cells. PLoS One. 2014; 9(6): e99525.
9. Lee Y, Ma J, Lyu H, Huang J, Kim A, Liu B. Role of erbB3 receptors in cancer therapeutic resistance. Acta Biochimica et Biophysica Sinica (Shanghai). 2014; 46(3): 190-198.
10. Koumakpayi IH, Diallo JS, Le Page C, Lessard L, Gleave M, Bégin LR, Mes-Masson AM, Saad F. Expression and nuclear localization of ErbB3 in prostate cancer. Clinical Cancer Research. 2006; 12(9): 2730-2737.
11. Vexler A, Lidawi G, Loew V, Barnea I, Karaush V, Lev-Ari S, Shtabsky A, Ben-Yosef R. Anti-ERBb4 targeted therapy combined with radiation therapy in prostate cancer. Results of in vitro and in vivo studies. Cancer Biology and Therapy. 2008; 7(7): 1090-1094.
12. Ben-Yosef R, Sarid D, Vexler A, Lidawi G, Inbar M, Marmor S, Starr A, Yaal Hahoshen N. Nuclear and cytoplasmic expression of ErbB-4 in prostate cancer. The International Journal of Biological Markers. 2007; 22(3): 181-185.
13. Samaan S, Lichner Z, Ding Q, Saleh C, Samuel J, Streutker C, Yousef GM. Kallikreins are involved in an miRNA network that contributes to prostate cancer progression. The Journal of Biological Chemistry. 2014; 395(9): 991-1001.
14. Darrington RS, Campa VM, Walker MM, Bengoa-Vergniory N, Gorrono-Etxebarria I, Uysal-Onganer P, Kawano Y, Waxman J, Kypta RM. Distinct expression and activity of GSK-3α and GSK-3β in prostate cancer. International Journal of Cancer. 2012; 131(6): E872-E883.
15. Lu L, Zeng H, Gu X, Ma W. Circulating tumor cell clusters-associated gene plakoglobin and breast cancer survival. Breast Cancer Research and Treatment. 2015; 151(3): 491-500.
16. Lai YH, Cheng J, Cheng D, Feasel ME, Beste KD, Peng J, Nusrat A, Moreno CS. SOX4 interacts with plakoglobin in a Wnt3a-dependent manner in prostate cancer cells. BMC Cell Biology. 2011; 12: 50.
17. Naito M, Komohara Y, Ishihara Y, Noguchi M, Yamashita Y, Shirakusa T, Yamada A, Itoh K, Harada M. Identification of Lck-derived peptides applicable to anti-cancer vaccine for patients with human leukocyte antigen-A3 supertype alleles. British Journal of Cancer. 2007; 97(12): 1648-1654.
18. Goldenburg-Furmanov M, Stein I, Pikarsky E, Rubin H, Kasem S, Wygoda M, Weinstein I, Reuveni H, Ben-Sasson SA. Lyn is a target gene for prostate cancer: sequence-based inhibition induces regression of human tumor xenografts. Cancer Research. 2004; 64(3): 1058-1066.
19. Zardan A, Nip KM, Thaper D, Toren P, Vahid S, Beraldi E, Fazli L, Lamoureux F, Gust KM, Cox ME, Bishop JL, Zoubeidi A. Lyn tyrosine kinase regulates androgen receptor expression and activity in castrate-resistant prostate cancer. Oncogenesis. 2014; 3: e115.
20. Sumitomo M, Ohba M, Asakuma J, Asano T, Kuroki T, Asano T, Hayakawa M. Protein kinase C delta amplifies ceramide formation via mitochondrial signaling in prostate cancer cells. The Journal of Clinical Investigation. 2002; 109(6): 827-836.
21. Fujii T, Garcia-Bermejo ML, Bernabó JL, [Caamaño J](http://www.ncbi.nlm.nih.gov.libaccess.lib.mcmaster.ca/pubmed/?term=Caama%C3%B1o%20J%5BAuthor%5D&cauthor=true&cauthor_uid=10713064), Ohba M, Kuroki T, Li L, Yuspa SH, Kazanietz MG. Involvement of protein kinase C delta (PKCdelta) in phorbol ester-induced apoptosis in LNCaP prostate cancer cells. Lack of proteolytic cleavage of PKCdelta. The Journal of Biological Chemistry. 2000; 275(11); 7574-7582.
22. Timofeeva OA, Zhang X, Ressom HW, Varghese RS, Kallakury BV, Wang K, Ji Y, Cheema A, Jung M, Brown ML, Rhim JS, Dritschilo A. Enhanced expression of SOS1 is detected in prostate cancer epithelial cells from African-American men. International Journal of Oncology. 2009; 35(4): 751-760.
23. Fizazi K. The role of Src in prostate cancer. Annals of Oncology. 2007; 18(11): 1765-1773.
24. Agoulnik IU, Vaid A, Bingman WE 3^rd^, Erdeme H, Frolov A, Smith CL, Ayala G, Ittmann MM, Weigel NL. Role of SRC-1 in the promotion of prostate cancer cell growth and tumor progression. Cancer Research. 2005; 65(17): 7959-7967.
25. Fu D, Liu B, Zang LE, Jiang H. MiR-631/ZAP70: A novel axis in the migration and invasion of prostate cancer cells. Biochemical and Biophysical Research Communications. 2016; 469(3): 345-351.
26. The UniProt Consortium. UniProt: a hub for protein information. Nucleic Acids Research. 2015; 43: D204-D212.
